# Genomic analyses of *Symbiomonas scintillans* show no evidence for endosymbiotic bacteria but does reveal the presence of giant viruses

**DOI:** 10.1371/journal.pgen.1011218

**Published:** 2024-04-01

**Authors:** Anna Cho, Gordon Lax, Samuel J. Livingston, Yumiko Masukagami, Mariia Naumova, Olivia Millar, Filip Husnik, Patrick J. Keeling

**Affiliations:** 1 Department of Botany, University of British Columbia, Vancouver, British Columbia, Canada; 2 Okinawa Institute of Science and Technology Graduate University, Okinawa, Japan; Virginia Tech: Virginia Polytechnic Institute and State University, UNITED STATES

## Abstract

*Symbiomonas scintillans* Guillou et Chrétiennot-Dinet, 1999 is a tiny (1.4 μm) heterotrophic microbial eukaryote. The genus was named based on the presence of endosymbiotic bacteria in its endoplasmic reticulum, however, like most such endosymbionts neither the identity nor functional association with its host were known. We generated both amplification-free shotgun metagenomics and whole genome amplification sequencing data from *S*. *scintillans* strains RCC257 and RCC24, but were unable to detect any sequences from known lineages of endosymbiotic bacteria. The absence of endobacteria was further verified with FISH analyses. Instead, numerous contigs in assemblies from both RCC24 and RCC257 were closely related to prasinoviruses infecting the green algae *Ostreococcus lucimarinus*, *Bathycoccus prasinos*, and *Micromonas pusilla* (OlV, BpV, and MpV, respectively). Using the BpV genome as a reference, we assembled a near-complete 190 kbp draft genome encoding all hallmark prasinovirus genes, as well as two additional incomplete assemblies of closely related but distinct viruses from RCC257, and three similar draft viral genomes from RCC24, which we collectively call SsVs. A multi-gene tree showed the three SsV genome types branched within highly supported clades with each of BpV2, OlVs, and MpVs, respectively. Interestingly, transmission electron microscopy also revealed a 190 nm virus-like particle similar the morphology and size of the endosymbiont originally reported in *S*. *scintillans*. Overall, we conclude that *S*. *scintillans* currently does not harbour an endosymbiotic bacterium, but is associated with giant viruses.

## Author summary

Endosymbiotic bacteria are found in a wide variety of hosts across the tree of eukaryotes and have been proposed to be evolutionarily and ecologically significant, but in most cases, we know little to nothing about them. This is exemplified by the stramenopile flagellate *Symbiomonas scintillans*, where the bacterial endosymbiont that gave the genus its name remains unidentified and has no known function. Here we used multiple genomic sequencing methods on two strains of *S*. *scintillans*, and showed absence of the endobacteria belonging to common endosymbiotic lineages. Instead, we identified giant viruses similar to those infecting prasinophyte green algae. Although further experiments are needed to verify the nature of the viral association with *S*. *scintillans*, our study is reminiscent of how the first mimivirus (named for mimicking gram-negative bacteria) was discovered, and we speculate similar discoveries will follow with ever-increasing genomic data of protists.

## Introduction

Understanding the evolutionary history of eukaryotes is inherently linked to understanding their symbiotic relationships with prokaryotes, whether it is in the form of genetically integrated organelles or the multitude of short-term endosymbioses with bacteria or archaea. Most of our understanding about the effects of endosymbiosis on eukaryotic evolution comes from the origin of mitochondria and plastids, and their involvement in eukaryotic diversification [1–4]. However, the impact of prokaryotic symbioses goes far beyond these rare organellogenesis events, given the diverse nature of symbioses affecting hosts in different ways [reviewed in 5,6]. Through genome sequencing, prokaryotic symbionts were found to be associated with all major eukaryotic supergroups, involved in a myriad of functions such as metabolism [5,7–9], defense [10], parasitism [11,12], and motility [10,13]. Additionally, some bacterial lineages have evolved to be “professional symbionts” [6] such as Chlamydiae, Rickettsiales, and Holosporales, consisting entirely of obligate endosymbionts or intracellular parasites of eukaryotic hosts [6,14–16].

Despite these impacts, most prokaryotic symbionts of eukaryotes are poorly studied, generally only observed with microscopy, and left unidentified and uncharacterized. For example, the only known case of prokaryotic endosymbiosis in non-phototrophic stramenopiles is found in the tiny (~1.4 μm) bikosia, *Symbiomonas scintillans*, where two geographically distinct strains were reported to harbour up to 6 endobacteria, and which served as the inspiration for its genus name [17]. The location of these endobacteria within the endoplasmic reticulum was of particular interest, as this is where plastids of phototrophic stramenopiles are located [17,18]. However, the identity and functional role of these apparent endobacteria has not been further investigated. To identify the endobacterium and its role in such a small protist, we conducted Fluorescent *in situ* hybridization (FISH) targeting various groups of bacteria and generated amplification-free shotgun metagenomics and whole genome amplification sequencing data of two strains of *S*. *scintillans*. This showed the absence of endobacteria of known endosymbiotic lineages. Instead, we observed a viral-like particle by transmission electron microscopy (TEM) and recovered three draft viral genomes related to prasinoviruses, namely nucleocytoplasmic large DNA viruses (NCLDVs) belonging to a member of the Phycodnaviridae family [19,20]. During the course of this work, one strain apparently lost the virus, while the other strain perished, so we were unable to conduct further experiments to verify the nature of the viral association. This work underscores how much is still unknown about endosymbioses, particularly in small heterotrophic protists. We expect that viral association is especially relevant to nano- or pico-eukaryotes, as there may simply not be enough space for endobacteria, and predict more such findings in the future.

## Materials and methods

### Culture collection and maintenance

All strains of *S*. *scintillans* used in this study are summarized in S1 Table, with the initial isolation dates and locations, sequencing methods, dates, and locations, and the culture collection centres. Briefly, two *S*. *scintillans* culture strains RCC257 and RCC24 were obtained from the Roscoff culture collection (RCC, France) on March 7^th^, 2022. The cultures were grown and maintained in 0.22 μm filtered and autoclaved marine f/2 media (30 PSU) with an autoclaved rice grain at the University of British Columbia (UBC), Canada. The cultures were kept in a 20°C incubator with a 12:12 h light:dark cycle and sub-cultured every two weeks in 30 mL. Using glass micropipettes, approximately 50 to 100 cells from each strain were collected and stored in 5 μL PCR-grade water after two rounds of rinsing in PCR-grade water on April 6^th^, 2022. The isolated cells were immediately subjected to three rounds of freeze-thaw cycles to promote lysis and stored at -80°C until whole genome amplification (WGA). Upon receiving the two strains, they were slow to grow (low culture density and no noticeable movement) and within 2 months of receipt, the strain RCC24 showed reduced viability and was eventually lost. This was also observed in the RCC, when their cultures perished with no identifiable cause at a similar time (M. Gachenot, assistant engineer/curator of RCC, personal communication, Oct 12^th^, 2022). In contrast, the strain RCC257 became denser and more active between the first round of cell collection in April 2022 and the second round of cell collection on June 28^th^, 2022 (we later suspected this boost of culture viability can be due to resistant cells or loss of viruses–see below). As a result, we also collected 50 cells from strain RCC257 on June 28^th^, 2022, for an additional WGA (hereafter, referred to as RCC257-late).

Independently at Okinawa Institute of Science and Technology (OIST), Japan, the culture strains RCC257 (hereafter referred to as RCC257-jp) and NIES-2589 (a strain synonymous to RCC24) were obtained from the RCC in December 2022, and the Microbial Culture Collection at the National Institute for Environmental Studies (NIES Collection, Tsukuba, Japan) in March 2021. Strain NIES-2589 will be hereafter referred to as RCC24-jp. RCC24-jp was cryopreserved at -160°C and was thawed in f/2 medium with an added rice grain. The RCC24-jp cultures were maintained in the same condition as above except with a 10:14 h light:dark cycle, and further processed for amplification-free shotgun metagenomics (AF-SMG; see Library preparation and sequencing). Strain RCC257-jp was grown in 20 μm filtered and autoclaved seawater with rice. All cultures were sub-cultured every 4 weeks.

### Library preparation and sequencing

Two strains of *S*. *scintillans* (RCC24 and RCC257) maintained at UBC were subject to WGA sequencing and one strain RCC24-jp, maintained at OIST was subject to amplification-free shotgun metagenomic sequencing. To prepare a WGA library of the isolated cells, a 4BB^TM^ TruePrime Single Cell WGA Kit was used following a manufacturer’s protocol with 12 h incubation at 30°C for the amplification reaction step. The amplified product was then cleaned with AMPure XP beads (Beckman Coulter, US), following a protocol described in the Nanopore Ligation Sequencing Kit protocol (SQK-LSK110, Oxford Nanopore Technologies, UK). Library preparation for WGA sequencing followed the Illumina DNA Preparation kit (Illumina, US) which uses a Bead-linked Transposome complex, resulting in ~350 bp library constructs. The WGA sequencing was performed on a NextSeq (mid-output) platform with 150 bp paired-end library constructs at the UBC Sequencing and Bioinformatics Consortium (Vancouver, Canada). Whole genome amplification sequencing was repeated twice using the same library constructs. For downstream analysis, the transcriptome of RCC257 (NCBI SRA accession number SRR24392496) was also used, which was prepared from approximately 20 isolated cells from the same sub-culture, described in Cho et al [21]. To minimize culture-associated bacterial reads, only single-cell isolated transcriptomes were used, as opposed to cDNA prepared from whole-culture RNA extract.

For amplification-free shotgun metagenomic (AF-SMG), 10 mL of RCC24-jp culture was filtered through a 5 μm syringe filter for enrichment (removal of large bacteria) followed by DNA extraction using the MasterPure Complete DNA and RNA Purification kit (Lucigen, US). The DNA extractions were prepared from multiple subsequent subcultures (in March, May, June, and October 2022). The AF-SMG libraries were prepared with the NEBNext Ultra II DNA Library Prep Kit for Illumina (NEB, US) and sequenced by the OIST Sequencing Centre using the Illumina MiSeq platform with 300 bp paired-end reads.

All the strain information with sequencing methods is summarized in S1 Table. The raw genomic data for this study is deposited in the NCBI Sequence Read Archive (SRA) with the accession numbers SRR26451788-SRR26451790, SRR26412500-SRR26412501, and SRR26943481, under the BioProject PRJNA1029166.

### Sequence processing: Assemblies and sub-assemblies of viral reads

The quality of raw sequencing reads for amplification-free shotgun metagenome, WGA, and transcriptome data were all examined using FastQC v0.11.9 [22]. The transcriptomic data were processed as described in Cho et al. [21]. Briefly, to correct random sequencing errors of the raw data, *k-mer* based Rcorrector (v3) [23] was used followed by Trimmomatic v0.39 [24] to remove transposase-inserts, SmartSeq2 primers, adaptors, IS-primers from library preparation and, low-quality reads (-phred33 LEADING:3 TRAILING:3 SLIDINGWINDOW:4:15 MINLEN:36). Error-corrected and trimmed forward, reverse and unpaired transcriptome reads were then *de novo* assembled using rnaSPAdes v3.15.1 [25]. To screen taxonomic affiliation of the contigs, we used BlobTools v2.3.3 [26,27], which incorporates NCBI nucleotide (nt) (using megaBLAST) [28] and UniProt reference databases [29], (using diamond BLASTX) both with e-value cut-offs at 1e^-25^ (—taxrule bestsumorder). We then manually removed contigs assigned to bacteria (free-living lineages) and obazoans. The open reading frames (ORFs) of the cleaned transcriptomes were then predicted with TransDecoder v5.5.0 [30]. The raw shotgun metagenome and WGA sequencing data were trimmed as described above without Rcorrector step, with corresponding adaptors and primers removed. The trimmed WGA reads from the three rounds of sequencing runs were then co-assembled using SPAdes v3.15.1 [31–33] with—sc and—phredoffset33 options. The same assembly parameters were used for the shotgun metagenome reads. For initial taxonomic and coverage screenings of the assembled transcriptomes, shotgun metagenome, and WGA assemblies, particularly to search for reported endobacteria, BlobTools was used to visualize search results of assemblies against NCBI nt (using megaBLAST) and UniProt reference databases (using diamond BLASTX), both with e-value cut-offs at 1e^-25^ (—taxrule bestsumorder). After failing to detect any obvious taxonomic signatures of endobacterial origin in both transcriptome and genomic data, a subset of WGA reads was reassembled by filtering reads with GC content below 40% (a common range for endosymbionts) and coverage above 10^25^ using SeqKit v2.3.0 [34], and assembling those with both SPAdes and Unicycler v0.5.0 [31,35].

With the initial BlobTools screening indicating the presence of prasinovirus taxonomic assignments in WGA sequencing data, trimmed WGA contigs were searched against the Reference Viral Database (RVDB) [36] using blastn (e-value cut-off 1e^-10^) followed by protein domain searches using hmmsearch (HMMER3.3) (hmmer.org) against virus orthologous groups (VOGs) (vogdb.org), Pfam, and giant VOG (GVOG) HMM databases compiled in ViralRecall [37]. The open reading frames (ORFs) were predicted using Prodigal-gv [38]. Contigs with the final ViralRecall scores above 10 were considered of viral in origin. For those with the final ViralRecall scores less than 10, if the number of VOG hits were higher than 3 and Pfam hits at the same time, we also considered these contigs to be viral. Additionally, all the contigs mapping to 16 prasinovirus genomes (BIIV, BpVs, OlVs, OtVs, OmVs, and MpVs) (S2 Table) using DNAdiff v1.3 [39] were kept. Contigs with viral hits from NCBI nt, clustered RVDB (RVDB-c), UniProt blast searches were kept excluding circular elements. These select contig results were cross-validated with blastx and blastn searches among VOG, RVDB, and diamond viral databases. The same searches were repeated on amplification-free shotgun metagenomic data, WGA data from RCC257-late, NIES-2589 (RCC24-jp), and ORF-predicted transcriptome data however, neither prasinovirus nor NCLDV reads were detected. Aside from microscopic observation, to confirm the absence of green algae contamination in the culture, we searched for small subunits of ribosomal RNA (SSU rRNA) in all sequencing data using barrnap v0.9 (32). We also carefully screened eukaryotic reads from the initial BlobTools results and found no evidence of green algae and other eukaryotic protist contamination.

An extracted subset of WGA viral contigs (707 contigs out of 69,958) was reassembled using SPAdes v3.15.1 with–sc,—careful and–phredoffset33 options, resulting in 748 scaffolds. Scaffolds with lengths under 100bp were removed. Additionally, blastn searches were repeated against NCBI-nt and RVDB databases to remove bacteriophage reads, resulting in 543 scaffolds with the total length of 469,314bp with 37.31% GC content. These filtered subset assemblies are hereafter referred as “viral-subset-scaffolds”. The viral-subset-scaffolds were then further scaffolded using 16 prasinovirus genomes as a guide with homology-based RagTag v2.1.0 [40,41]. This reference-guided assembly method does not alter the scaffold sequences but reorients and reorders them by aligning to a reference genome, creating a single scaffold or a pseudomolecule. The pseudomolecule or the single scaffold of viral metagenomically assembled genomes will be hereafter referred to as vMAG. Out of 543 viral-subset-scaffolds, 279 were recruited for the assembly of 16 vMAGs. The remainder of the 264 scaffolds were not recruited to any reference genomes despite having 194 out 264 scaffolds with ≥80% similarity to known sequence identities (ID) and e-value < 1e^-25^ hit to prasinovirus (the rest of the scaffolds had lower % ID or no hits to the database). This is due to the majority of the scaffolds (215/264) being shorter than 500bp, which were filtered out due to the small alignment length threshold (1000 bp). Additionally, a pre-defined *k*-mer and window size (19 bp) in read mapping to the reference genomes may have affected correct scaffold placements of sequence variants in these potentially new vMAGs.

The completeness of each reference-guided assembly was assessed using CheckV v0.8.1 with CheckV-db v1.5 [42] (S2 Table). The assembled vMAGs with the highest completeness and the corresponding reference genomes are circularized for visualization with BLAST Ring Image Generator (BRIG) v3 [43].

### Draft viral genome annotation and gene content comparison

The ORFs for each reference guided vMAGs (= pseudomolecules) were predicted with Prodigal-gv and further annotated with Prokka and ViralRecall (scores > = 10) (S1 Table and S1 Dataset). The same was repeated with the 16 prasinovirus genomes (S2 Table). To compare shared orthologs among vMAGs and published viral genomes, all the ORFs were used in all-versus-all blastx search [44]. The blastx (e-value = 1e^-5^ and query-cover = 50) result was then clustered first by 95% similarity using CD-hit v4.8.1 [45] followed by MCL algorithm (inflation = 2). Only clusters with hits from a minimum of three different genomes (including vMAGs) were retained (432 clusters). Amino acid sequences of each cluster were then aligned (MAFFT v7.481 [46]) and trimmed (trimAl v1.2rev59 [47]), which was then used to build 432 HMMs. The resulting HMMs were then searched against individual reference and draft genomes using hmmsearch HMMER v3.3 (e-value 1e^-10^ and domain e-value 1e^-8^) to confirm the presence of the protein clusters in the genomes. The outcome of shared protein clustering hits for each genome was summarized in an upset plot (S3A Fig). All BpV- and BIIV-vMAGs were combined as “BV-vMAGs”, all OlV-, OmV-, and OtV-vMAGs were combined as “OV-vMAGs”. Similar grouping was done for published genomes that were used as a reference-guide to assemble vMAGs.

### Prasinovirus hallmark gene search and phylogeny construction

To construct a phylogenetic tree of prasinoviruses, we searched for 22 prasinovirus hallmark genes [48,49] in the predicted ORFs of WGA viral-subset-scaffolds using blastp (e-value 0.001) and hmmsearch (–E 1e-3 –domE 1e-3 –incE 1e-3 –incdomE 1e-3). Candidate genes from the predicted ORFs were then concatenated with the corresponding alignments and then realigned with MAFFT (—auto) and trimmed with trimAl (-gt 0.3 and -st 0.001). We then constructed a single gene tree for each of the prasinoviral hallmark genes using IQ-TREE v2.1.0 under the LG+G4 amino acid model with 1000 ultrafast bootstrap pseudoreplicates. Each single-gene tree and corresponding alignment were manually examined to discern viral paralogs and orthologs from cellular proteins. For some of the single-gene alignments, the candidate genes were manually merged if the gene fragments had overlapping regions and were positioned within the same clade. The 22 cleaned prasinovirus hallmark single-gene alignment were then concatenated, realigned with MAFFT, trimmed with trimAl, and a multi-gene phylogenetic tree was inferred using IQ-TREE v2.1.0 under the LG+G4+F model and 1000 ultrafast bootstrap pseudoreplicates. We searched for the same prasinovirus hallmark genes in predicted ORFs from our transcriptome data in the same manner, however, no hits were found.

The hallmark gene alignments, relevant intermediate files, gene-tree files, vMAG genome and protein sequences are uploaded on Dryad Digital Repository [50].

### Transmission electron microscopy (TEM)

To visualize a virus-like particle (VLP) in unfiltered strain RCC24, 5 μL of the culture was deposited onto glow-discharged (60 sec at 50 mA; Leica EM ACE200) formvar/carbon-coated 400 mesh copper TEM grids. Samples were stained with 2% uranyl acetate for 60 s. Excess UA was removed by gently placing a filter paper at the edge of the grids and subsequently transferred to a FEI Tecnai Spirit TEM (Thermo Fisher, USA) operating at 80 kV acceleration voltage. Images were captured with a DVC1500M camera and AMT Image Capture Engine V601 software (MA, USA). VLP diameter was measured with the AMT built-in measurement tool. All sample processing and TEM imaging were carried out in a sterile environment where no other viral experiments were done prior to the imaging.

### Fluorescence in situ hybridization (FISH)

For the RCC257 strain grown at UBC, Canada, 10 mL of culture was spun down in 15 mL centrifuge tubes at 3000 rpm, at 4°C for 10 min. The centrifuged cells were collected from the bottom of the tubes and transferred into 1.5 mL microcentrifuge tubes. Approximately 7 μL of the collected cells were placed on Poly-D-Lysine-coated glass slides (Sigma-Aldrich, US) and demarcated with a LiquidBlocker (Electron Microscopy Sciences, US). An equal amount of 4% paraformaldehyde (in water) was added to the slides. After all the liquid evaporated, 95% ethanol was added to the marked spot on the slides and incubated until complete drying. The slides were dipped in 50%, 80%, and 100% ethanol for 10 min each. The slides were then incubated overnight in a dark humidity chamber at 46°C with 10 μM of probe EUB338-Green prepared in a hybridization solution (1 M, pH 8.0 Tris HCl; 5 M NaCl, 1.3% SDS). The slides were gently rinsed twice in 48°C hybridization solution for 10 min, followed by 15 min rinse in water at room temperature. After completely drying liquid, 20 μL of SlowFade Gold with DAPI (Life Technologies, US) were added and visualized with an Olympus BX53 at the UBC Bioimaging Facility, Canada.

To verify the lack of endobacteria in sub-cultures grown in Japan, a separate FISH protocol was done on the RCC24-jp and RCC257-jp strains. Each of the 10 mL of culture were fixed with 3.2% formaldehyde at 20°C for 20 min and spun down at 4000 rpm at 4°C for 15 min. The centrifuged cells were washed with 1x PBS and seeded onto a 0.1% polyethyleneimine-coated 18 mm round coverslip (Matsunami Glass Ind., Ltd, Japan) in a 12-well plate. To allow attachment to the coverslip, the fixed cells were incubated for 3 h in 1x PBS. The attached cells were then washed three times each for 5 min in 1x PBS, 0.3% 1x PBS-Tx (0.3% Triton X-100, pH7.4), then in hybridization buffer (20 mM Tris-HCl; 30% formamide; 0.01% SDS). The fixed cells were hybridized with probes EUB338-Alexa488, EUB338-Alexa647 (Eubacteria), and CF319a-Alexa647 (Bacteroidetes) [0.1 μM] [51] (ThermoFisher Scientific, Japan) with DAPI [0.01 ug/mL] (Roche, Germany) and incubated overnight in a 42°C humidity chamber. For RCC24-jp, additional probes targeting Planctomycetes (PLA46) [52], alpha- (ALF969), and gamma-Proteobacteria (GAM42a) [53] were hybridized. To remove unbound probes and DAPI, the coverslip was gently rinsed three times in 0.3% 1x PBS-Tx solution for 5 min and twice in 1x PBS. After drying, the coverslip was mounted onto a glass slide with ProLong^TM^ Diamond Antifade Mountant (ThermoFisher Scientific, Japan) and incubated at room temperature overnight in the dark. The hybridized sample was kept at 4°C in the dark until visualization on Leica TCS SP8 Inverted Confocal Microscope at the OIST Imaging Facility (Okinawa, Japan). The brightness and contrast of all images were adjusted using ImageJ v1.53 and sharpness with Inkscape v1.2.1.

## Dryad DOI

10.5061/dryad.mw6m90644

## Results and discussion

### No bacterial sequences from known clades of common endosymbionts

To identify the symbionts of *Symbiomonas scintillans*, we sequenced two geographically distinct strains (RCC24 isolated from Pacific Ocean and RCC257 from the Atlantic Ocean) maintained under culture conditions. In most of our sequencing data, a large representation of the host sequence was found as expected. The exception to this is the WGA data from RCC24, where no host sequences could be identified (see below). Given the original description of this taxon suggested these symbionts were bacterial, we first searched for bacterial reads assigned to well-known endosymbiotic lineages such as Rickettsiales, Holosporales, or Chlamydiae in all the analyzed genomic and transcriptomic data. No such putative symbiont reads were found, and instead the bacterial reads were largely assigned to common environmental, or culture-associated Alphaproteobacteria, Gammaproteobacteria and Balneolia such as *Marinobacter* spp., *Epibacterium* spp., *Hyphomonas* spp., *Zhongshania* spp., *Balneola* spp., and *Labrenzia* spp. (S1A–S1C Fig). When sequences that had no taxonomic affiliation in WGA data were removed, a scaffold assigned to *Marinobacte*r *salinus* had the third highest coverage up to x95,000 (N50 = 116K), after the ones assigned to Oomycota (N50 = 276) and *Cafeteria roenbergensis* (up to x102,851 coverage, N60 = 63K), a species closely related to *S*. *scintillans* (S2 Dataset) [21]. Notably, *Marinobacter* spp., *Labrenzia* spp., and *Hyphomonas* spp. were all reported to be common in cultures of *Ostreoccocus tauri*, Symbiodiniaceae, *Alexandrium* spp., and discobids [54–60]. Accounting for this overwhelming representation of culture-associated bacteria, a subset of whole-genome amplification (WGA) data was selected and reassembled based on lower GC content, which is usually associated with endosymbionts. However, no sequences assigned to endosymbiotic bacterial lineages were detected. To account for unequal genomic amplification of WGA causing loss of AT-rich and local repeat regions, and secondary structures [61], we also searched bacterial reads in amplification-free shotgun metagenomic data. Many bacterial lineages with high-coverage in WGA were also found in the shotgun metagenomic data (e.g., *Marinobacter* spp., *Hyphomonas* spp., *Balneola* spp.) in addition to *Marinovum algicola* and a member of Phycisphaeraceae, but no known endosymbiotic lineages nor any draft bacterial genomes with “symbiotic features” such as small genome size, AT-rich content, or rapid sequence evolution could be identified in any of these data.

**Fig 1:**
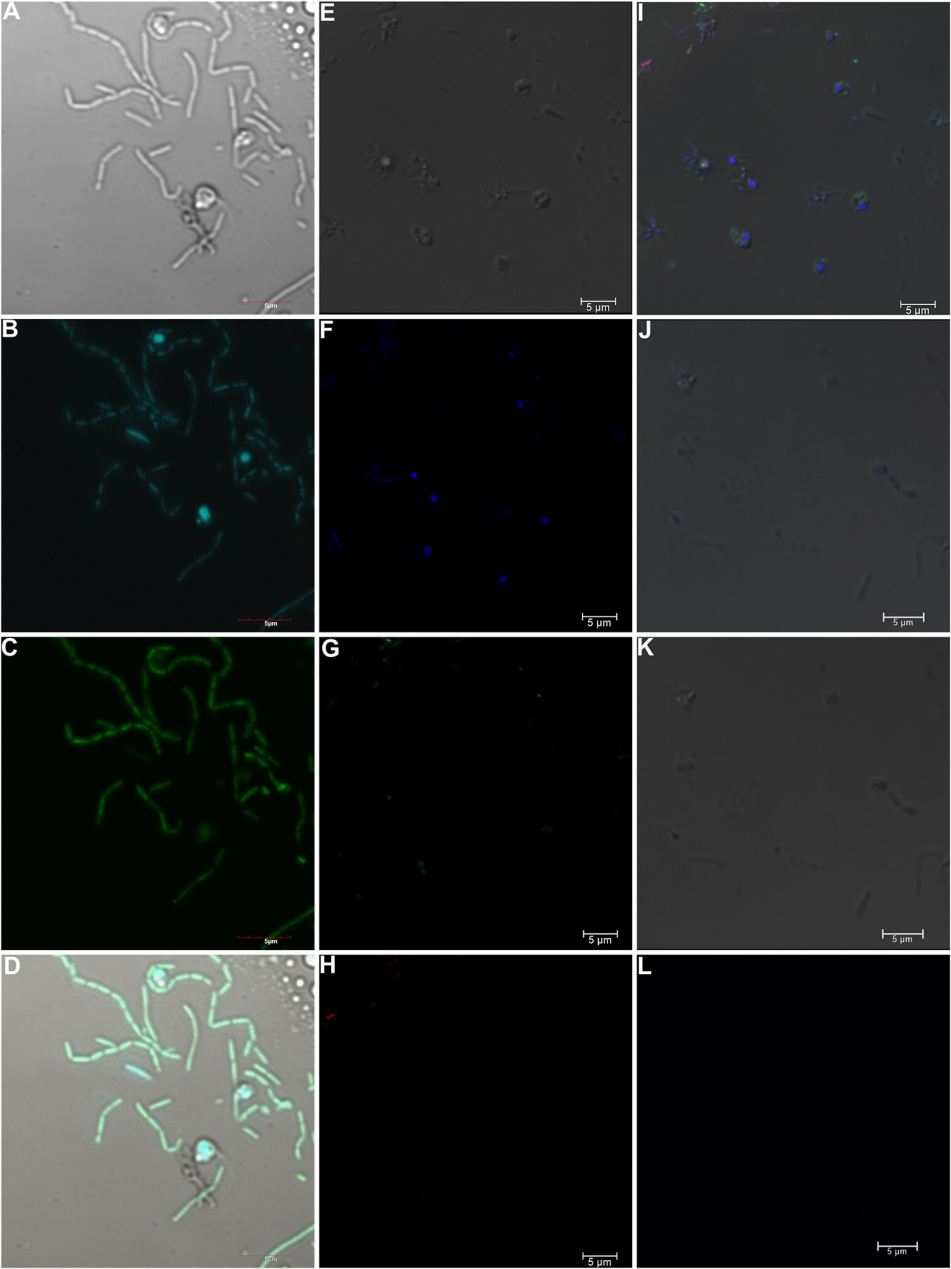
FISH analysis on *S*. *scintillans*. (A-D) RCC257 and (E-L) RCC257-jp showing no endobacterial signals. (A), (E) and (J) Brightfield; (B) and (F) DAPI; (C) and (G) EUB388 probe under 473 and 488 nm; (D) merged image of (A-C); (H) CF319 probe under 647 nm; (I) merged image of (E-H); (K) merged image of unstained DAPI, CF319 and (L) EUB388 images. Scale bars = 5 μm.

The absence of endosymbiotic bacteria in all the sequencing data was further supported by the absence of a bacterial signal using fluorescence *in situ* hybridization (FISH) of all sub-cultures of *S*. *scintillans* grown in Canada (RCC257) and Japan (RCC257-jp) (Fig 1). We observed the same trend in RCC24-jp (S2 Fig) using additional probes targeting Planctomycetes, Bacteroidetes, Alphaproteobacteria, and Gammaproteobacteria. In all our assembled WGA data, no sequences were assigned to Archaea while the amplification-free shotgun metagenome data had some Archaea contigs with low coverage (x1-7).

### Multiple prasinovirus-like vMAGs are associated with RCC257 and RCC2 24

Instead of endobacteria, we detected contigs assigned to prasinovirus with up to x200 coverage in RCC257 (S1 Fig and S2 Dataset). When viral-subset-scaffolds were re-assembled and further scaffolded using a reference-guide approach, we recovered three viral metagenomically assembled genomes (vMAGs) related to the prasinovirus genera, *Bathycoccus prasinos* virus 2 (BpV2), *Ostreococcus lucimarinus* virus 1 (OlV1), and *Micromonas pusilla* virus Pl1 (MpV_Pl1). The completeness of vMAGs were the highest for the BpV2-guided assembly (BpV2-vMAG), with 100% completeness. Among OV-guided and MpV-guided vMAGs, OlV-1-guided assembly (OlV1-vMAG) and MpV_Pl1-guided assembly (MpVPl1-vMAG) had the most completeness with 54% and 18%, respectively (S2 Table).

**Fig 2:**
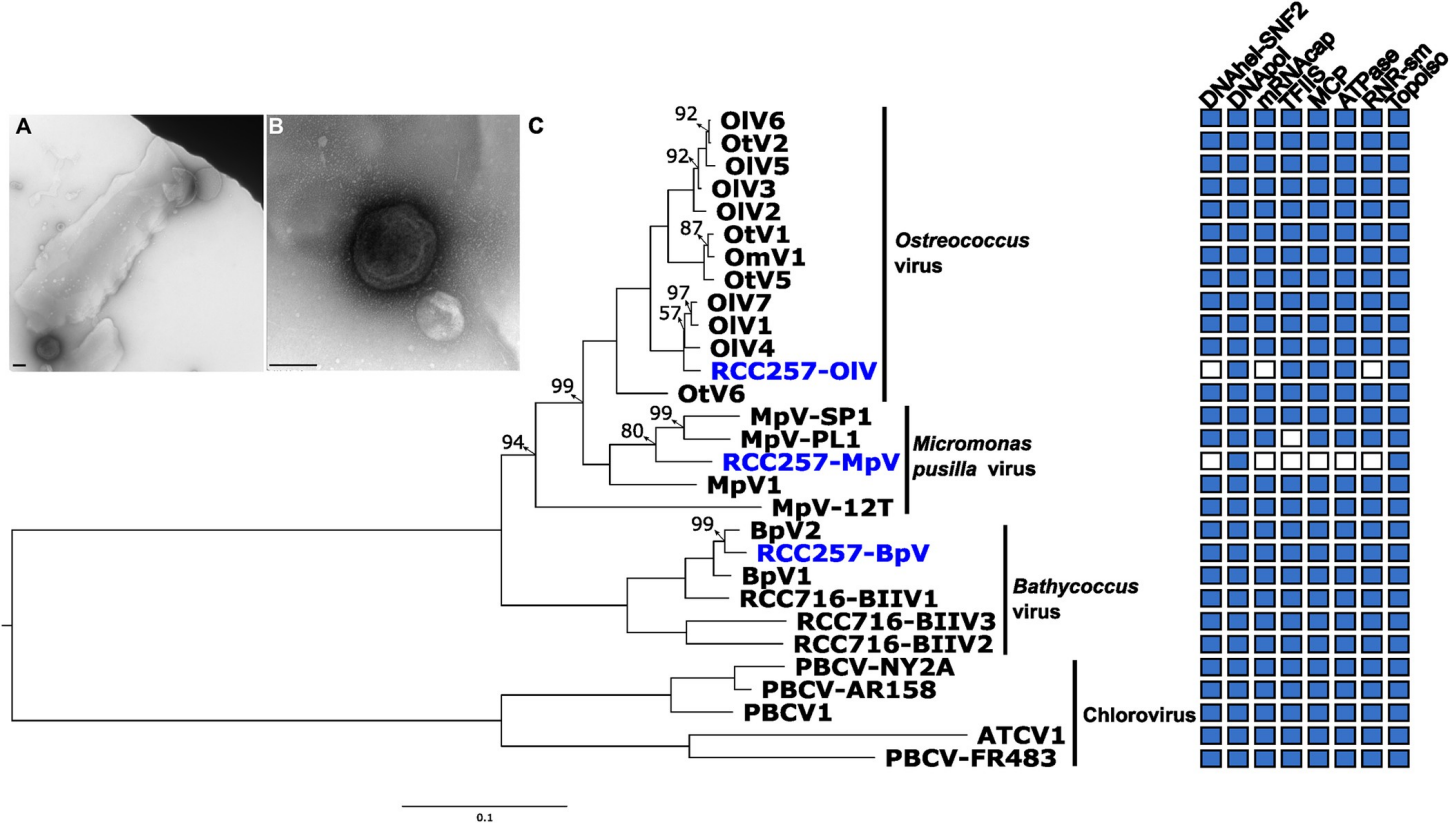
Prasinovirus multi-gene tree and a virus-like particle (VLP). (A) and (B) Detection of VLP in negatively stained RCC24. (B) Close up of the VLP in (A). The diameter of the VLP is 192 nm. Scale bars = 100 nm. (C) A multi-gene prasinovirus phylogeny reconstructed from 22 core genes (5,213 sites) using IQ-TREE2 LG+F+G4 model. The right panel shows presence-absence of select core genes. Single-copy genes are DNApol (DNA polymerase B), DNAhel-SNF2 (SNF2 helicase), mRNAcap (mRNA capping enzyme), ATPase, and RNR-sm (RNR small subunit). The tree is rooted with Chlorovirus (PBCVs and ATCV) for visualization. Only nodes <100% ultrafast bootstrap supports are labelled. OlV = *Ostreococcus lucimarinus* virus; OtV = *Ostreococcus tauri* virus; OmV = *Ostreococcus mediterraneus* virus; MpV = *Micromonas pusilla* virus; BpV = *Bathycoccus prasinos* virus; BIIV = *Bathycoccus* sp. virus clade BII. PBCV = *Paramecium bursaria chlorella* virus; ATCV = *Acanthocystis turfaceae chlorella* virus.

We compared the number of shared scaffolds and gene contents among BV-, OV-, and MpV-vMAGs to verify the presence of multiple different virus genomes. Only up to two recruited scaffolds were shared between vMAGs of BVs, OVs and MpVs (S3B Fig). When the shared orthologs were examined among all vMAGs using 16 reference genomes, we observed the same trend (S3A Fig). Multiple copies of single-copy-genes (e.g., DNA polB, DNA helicase, and mRNA capping enzyme) [62,63] were detected in viral-subset-scaffolds, each corresponding to three groups of prasinoviruses [63]. All 22 genes were placed within a BV clade, 9 genes in an OV clade, and 4 in a MpV clade (Fig 2). These results support the presence of multiple giant viruses, altogether referred as *S*. *scintillans* viruses (SsVs), rather than a single genome mapping to multiple reference genomes. In RCC24 we found no evidence of host reads (see above), but also found evidence for three giant viruses very similar to those found in RCC257 (S4 Fig). No prasinovirus reads were detected in RCC24-jp.

The presence of multiple viral species within a single host species is rare. However, multiple viral species were detected in three different species of Ectocarpales, a group of brown algal stramenopiles [64,65]. In these host species, up to two major capsid protein (MCP) genes of different Phaeoviruses (Phycodnaviridae) subgroups were found. One of these phaeoviruses (EfasV), can infect different genera of Ectocarpales [66]. Although prasinoviruses are reported to have a narrow host range at the strain or species level [48,67–70], the close relationship to phaeoviruses might indicate wider host range is also possible for these new prasinoviral vMAGs. Additionally, the name “prasinoviruses” likely reflects a sampling bias in the first reports, as is the case for many viruses. Notably, both *Monkeypox* (MPXV) [71] and *Cucumber mosaic viruses* (CMV) [72] were named after their first isolation from *Macaca fascicularis* (macaque monkeys) and *Cucumis sativus* (cucumbers), respectively, but MPXV was subsequently reported to infect other hosts including humans and squirrels (for MPXV) (reviewed in [73]), and CMV in legumes and ornamental plants [74].

### Genome characteristics of vMAGs

**Fig 3:**
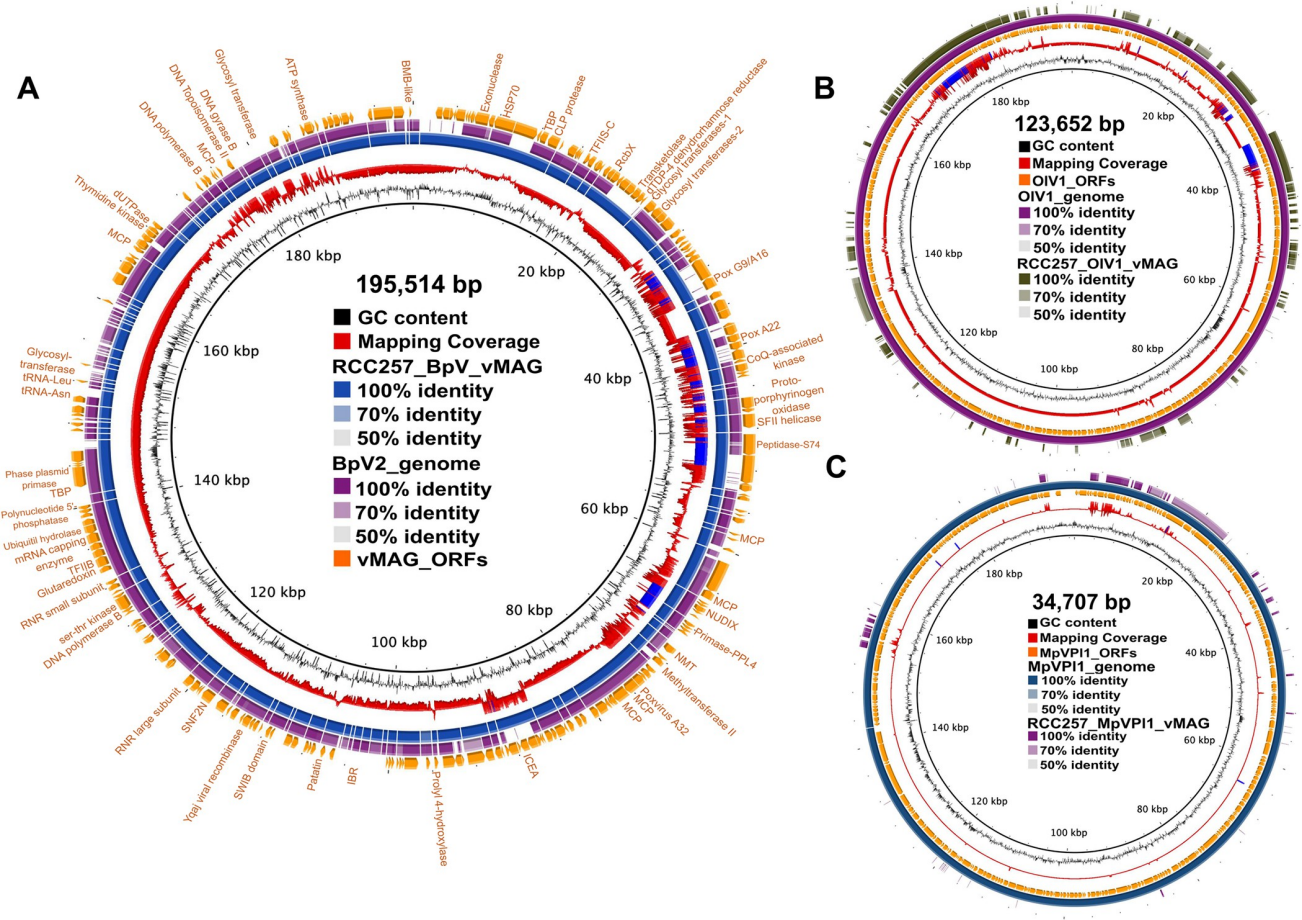
Genome overview and comparison of select vMAGs to corresponding reference genomes. Circularized representation of (A) RCC257 BpV-vMAG compared to BpV2 genome; (B) OlV1 genome compared to RCC257 OlV1-vMAG; (C) MpV_Pl1 genome compared to MpVPl1_vMAG, in an ordered set of coding sequences, represented by blocks shaded by similarity. The sizes of vMAGs labelled in the centre. (A) Mapping coverage is based on RCC257 BpV-vMAG mapped to viral-subset-scaffolds and regions with the coverage more than one standard deviation [62.1] from the mean coverage [50.8] are shown in blue spikes. The outermost ring represents predicted ORFs of the vMAG with manually annotated protein from Prodigal-gv and Viralrecall. (B) Mapping coverage is based on OlV1 genome mapped to viral-subset-scaffolds and regions with the coverage more than one standard deviation [8.4] from the mean coverage [3.1] shown in blue spikes. Only ORFs from the reference OlV1 genome is shown and the partial RCC257 OlV1-vMAG CDS are shown in the outermost ring. (C) Mapping coverage is based on MpV_Pl1 genome mapped to viral-subset-scaffolds and regions with the coverage more than one standard deviation [7.8] from the mean coverage [0.6] shown in blue spikes. Only ORFs from the reference OlV1 genome is shown and the partial RCC257 OlV1-vMAG CDS are shown in the outermost ring. See S2 Table for annotation in a tabular format.

While many genes and ORFs were predicted on all vMAGs (S1 Dataset), only BpV-vMAGs were fully annotated (Figs 3 and S5 and S1 Dataset). For BpV-vMAG, 297 ORFs were predicted, including homologues of Hsp70 (a known protein in BpVs with a green algal host origin [75]), DNA methyltransferase, and multiple MCPs were identified (S1 Dataset). For the OlV1-vMAG, 149 ORFs were predicted, while MpVPl1-vMAG had 47 ORFs predicted (S2 Table). OlV- and MpV-vMAGs from RCC24 had more complete assemblies (S5 Fig and S2 Table).

Compared to published prasinovirus genomes with 3–5 tRNAs (three for BpVs), only two tRNAs in RCC257 BpV-vMAG were predicted (Fig 3A): tRNA-Leu and tRNA-Asn. Similar to chloroviruses, four tRNAs were predicted in RCC24 BpV-vMAG, two of them being tRNA-Asn (S5 Fig) [75]. We detected five and six MCPs in RCC24 and RCC257 BpV-vMAGs, respectively, as was the case for BVII1-3 (Fig 3 and S1 Dataset) [48]. A high number of MCPs (up to nine) is unique to Phycodnaviridae, however, its implications in host entry or capsid assembly are currently poorly understood [63,75,76]. Along with other common prasinovirus proteins involved in carbohydrate synthesis (i.e., dTDP-4-dehydrorhamnose reductase, and five glycosyltransferases), we also detected ribulose-phosphate 3-epimerase in RCC257 BpV-vMAG (S1 Dataset), which was unique to BIIV-2 and -3 among prasinoviruses [48].

To evaluate unique gene contents in BV-vMAGs, we generated protein clusters and compared them between 16 vMAGs and reference genomes. We observed that 26 protein clusters were unique to BV-vMAG (including BpVs- and BIIVs-vMAGs) (S3A Fig and S1 Dataset). Although most of the annotation indicated HMM hits to hypothetical proteins of prasinoviruses, we detected a protein cluster assigned to 4-hydroxy-2-oxopentanoic acid aldolase. In prasinoviruses, this enzyme was only found in MpVs and is involved in biosynthesis of isoleucine, leucine, and valine that might be important in capsid formation [75,77]. Additionally, in both RCC24 and RCC257 BpV-vMAGs, we detected the IceA gene (“induced by contact with epithelium” endonuclease) gene, a putative virulence gene in *Helicobacter pylori* [78] which is also present in the *Chrysochromulina ericina* virus (Mimiviridae; NCLDVs) [79].

### SsV vMAGs are associated with *S*. *scintillans*

As prasinoviruses are known to be host-specific and have not yet been described in other hosts, we wanted to rule out the unlikely possibility that these new viruses came from a cryptic prasinophyte in the culture. We detected no green algal SSU sequences or signals indicative of green algal contaminants in any of the microscopic observation and sequencing data. In our WGA data, there were 15 scaffolds assigned mitochondrial genes of various Chlorophyta species (S2 Dataset), with read-counts ranging from 1 to 331. A close inspection of these scaffolds showed that these hits are likely not green algal contamination, as the taxonomic assignments were based on short read lengths. Additionally, some of the blastp hits of the same scaffolds indicated a stramenopile origin (Bikosia, ochrophytes, and oomycetes), suggesting these regions of the scaffolds are likely from the host and represent conserved homologs found in mitochondria across different eukaryotes. We observed similar patterns with scaffolds taxonomically identified as belonging to Rhodophyta (S2 Dataset).

The possibility that prasinoviruses contaminated the culture media is also highly unlikely, given both the sterilizing protocol (autoclaving, filtering, and UV sterilization) and single-cell isolation. These methods could hardly result in near-complete BpV-vMAGs from contaminant viruses, which require a minimum of 10^5^ VLP to reach the observed read depth [80]. Due to the loss of viral signals in RCC257-late and the complete loss of the RCC24 strain, we could not conduct an infection assay or purify lysates. However, given the sequence coverage of prasinoviral reads, completeness of BpV-vMAGs, lack of evidence of green algae in the cultures, and sample processing method, we argue that the SsV vMAGs are indeed directly associated with *S*. *scintillans*. This is further supported by the two similar but distinct strains of *S*. *scintillans* contained two similar but distinct sets of three giant virus genomes.

### TEM observation of a VLP

A virus-like particle (VLP) from RCC24 was visualized with negative stain TEM (Fig 2A and 2B). The VLP exhibited an icosahedral shape with a diameter of 192 nm, which is unusually large compared to previously characterized prasinoviruses [77]. However, it fell within the size range (180–240 nm) of the inclusion described as endobacteria in *S*. *scintillans* [17]. Indeed, the morphology of the “endobacteria” in the original description (see Fig 1D in [17]) closely matches that of the VLP in Fig 2A and 2B. We did not observe VLPs in the actively growing RCC257 strain, as expected as the NCLDV reads were no longer detected in RCC257-late.

The *S*. *scintillans* “endobacteria” were also described to be located within the endoplasmic reticulum (ER), which continues as perinuclear space of a nuclear envelope [17]. This location was emphasized to be potentially relevant for the origin of plastids in deep-branching lineages of stramenopiles and compared to the location of plastids found in photosynthetic lineage of stramenopiles [81,82]. However, the ER is also a site for viral protein glycosylation [83], membrane protein folding [84], genome replication, and pre-capsid assembly [85,86]. Within the Phycodnaviridae, the development of a Phaeovirus infecting *Hinckisa hinckisae* has been observed within the ER, in which viral capsids are derived from the ER membrane [19,87].

### Possible nature of associations: Endobacteria, SsVs, and *S*. *scintillans*

Two decades have passed since the original description of *S*. *scintillans*, and the present analysis, raising many questions about how to connect data from the original description with data currently at hand. There is no direct evidence to verify the exact nature of the association between SsVs and *S*. *scintillans* and similarly, there is no way to equate the SsVs to the intracellular inclusions described in 1999. Because our experimental design was to identify endobacteria and because there is no longer any living host-virus pair in culture, experiments such as infection assays, virus-targeted FISH or PCRs, or thin-section TEM to show virus particles within the cells are not possible. At the same time because there was no sequence data associated with the original genus or endobacteria description, we cannot compare the current data directly with any data from the original description.

There are several possible explanations that formally account for the data, and we will review them here. First, it is possible that inclusions originally described are endobacteria that are still present, but were not detectable in genomic analyses, or belong to one of the normally free-living lineages we did detect. This is not readily consistent with the FISH data, however, and is also not consistent with the genomic observations from most other bacteria endosymbionts of protists [6].

Second, it is also possible the endobacteria were lost and the viruses were acquired later. The idea that the endobacteria may have been lost is not without precedent, since this has been observed in previous cultures [88], but how multiple viruses could have been gained is a much more difficult problem. The read-depth in the vMAG assemblies suggests viral DNA was highly represented in these cultures, and by extension that these viruses were replicated in the cultures. Since no other eukaryotes were in the cultures, it also suggests the viruses were most likely replicating in *S*. *scintillans* (since the viruses need some host and no other eukaryote is evident). Therefore, for the viruses to have been gained after the original description, the two cultures would have to have been exposed to two related but distinct sets of viruses that could each infect and replicate in *S*. *scintillans*.

Third, it is also possible the viruses have been endogenized within the host genome [37,63,89]. This is not obviously consistent with absence of viral reads in some of the sequencing data (S1 Table) or the TEM evidence for viral particles. We also examined this possibility using ViralRecall [37], which did not detect viral regions with potential host sequences flanking viral contigs.

Lastly, it is possible that the initially reported endobacteria are actually giant viruses. This possibility is consistent with all the sequencing and FISH analyses, but contrary to the identification of the inclusions made in the original description based on thin-section TEM. However, when this was observed, the field of giant viruses was relatively young, so the only logical identification of a large inclusion in the ER would be a bacterium. In retrospect, many of these TEMs actually resemble giant virus particles, and we observed an extracellular VLP that falls within a similar size range and resembles a shape of the reported endobacteria (compare Fig 2A and 2B with Fig 1D of [17]). However, as noted above since the cultures are now gone and the data are generally non-overlapping, this possibility can obviously not be verified either.

Another complication with the last possibility is in how to explain the long-term persistence of viruses in these cultures, in particular as it must have been followed after 20 years by a sudden loss of viruses (RCC257-late) or the death of the strain (RCC24). One *O*. *mediterraneus* culture with a decade-long co-existence with OmV2 was found to be a co-culture of resistant (R) and susceptible (S) strains, where the host showed two reversible phenotype phases that are thought to explain the long-term stability of the system [90,91]. It was hypothesized that the RS-switching may be a common long-term strategy for other NCLDVs-affected hosts, and persistent infection is a known strategy for phaeoviruses, a close relative of prasinoviruses [19,92,93]. Some resistant hosts have been observed to produce infective viruses with nearly undetectable low transcription and without typical lytic events [90,94], reminiscent of the fact that no prasinovirus reads and hallmark genes were detected in our RCC257 transcriptome data (and also to Herpesvirale [95], another dsDNA virus distantly related to NCLDVs). Interestingly, when susceptible and different types of resistant cells (R^P^ vs. R^NP^: viral-producing vs. non-producing) were cloned and co-cultivated, the viruses were eventually eliminated in the co-cultivated R^P^ and R^NP^ culture while, susceptible cells became dominant in the S and R^NP^ co-cultivated culture [94].

To examine the possibility that virophages are involved in the host-virus dynamic, we searched for virophage genes or virophage-like elements (VLEs) [96,97] in the initial assembly without taxonomically filtering scaffolds, due to the nature of some virophage genes being recombinant, horizontally transferred, or homologs that are shared with cellular organisms or transposable elements (i.e., polintons), and NCLDVs [97]. We detected OLV2 (an uncharacterized protein) only in RCC257 WGA, forming a sister lineage to Yellowstone Lake virophage 1 (YSLV1) [98]. Although this result is insufficient to conclude the involvement of virophages or VLEs in our data, deeper sequencing and assembly of the *S*. *scintillans* genome could potentially verify the presence and nature of virophages or VLEs association.

We suspect the lack of prasinovirus reads in RCC24-jp is due to long-term cryopreservation. For example, in the *Paramecium bursaria chlorella* virus (PBCV-1), the strength of infectivity decreased upon cryopreservation and more so if the samples were frozen shortly after post-infection [99,100]. Whether our observation is based on differences in host strains or SsVs, or a combination of both, characterization of host genomes along with further searches of prasinovirus in non-Mamiellophycean hosts will provide insights into the dynamics of persistent infection.

The current observations echoes the first discovery of the mimivirus, which was initially described as “Chlamydia-like obligate parasites” in an amoeba [101]. It took six years to correctly characterize the parasites as Mimivirus [102]. Conversely, the bacterium *Chromulinavorax destructans* [103] was recently been described as a bacterial parasite of *Spumella elongata* (a photosynthetic stramenopile), but it was initially studied as a putative giant virus, due to a replicating morphology resembling a viral factory of some giant viruses. Both these cases illustrate how difficult it can be to identify the nature of an intracellular symbiont, suggesting that more studies on the diversity of symbioses in heterotrophic nano- or pico-flagellates should yield more such surprises and taxonomic re-assignments of many symbionts will also follow.

## Supporting information

S1 Table Summary of different strains and sequencing data of *Symbiomonas scintillans* examined in this study. RCC = Roscoff Culture Collection, France; NIES = Microbial Culture Collection at the National Institute of Environmental Studies, Japan; WGA = Whole genome amplification; AF-SMG = Amplification-free shotgun metagenome.
(DOCX)

S2 Table Summary of genomic characteristics of prasinoviruses and subsequent vMAGs from RCC24 and RCC257. CheckV% indicates completeness for each vMAG assemblies.
(DOCX)

S1 Fig Summary of BlobToolKit analysis of unfiltered WGA assemblies of RCC257 (top row) and RCC24 (bottom row). (A) Blob plots based on mean coverage (per-base) in y-axis and mean GC contents in x-axis. Each “blob” represents a square-root scaled size (showing max size) of a scaffold with its size representing the length or span. The blobs are coloured according to the top ten taxonomic assignment at the genus level (‘bestsum’ taxrule), based on coverage. Sum lengths along each axis are plotted on histograms. All reads assigned to prasinoviruses are highlighted with purple squares around each blob. (B) Snail plots visualizing quality of the initial assembly represented by N50 and N90. The purple squares in the blob plots and ones positioned at the outermost part of the plots are scaffolds assigned to prasinoviruses. (C) Histograms showing coverage (y-axis) for top ten genus (including “no-hit”, “undefined” and “others”).
(PDF)

S2 Fig FISH analysis on *S*. *scintillans* RCC24-jp showing no endobacterial signals. (A), (F), (K), and (P) Brightfield; (B), (G), and (L) DAPI; (C) CF319 probe under 647 nm; (D) and (M) EUB388 probe under 488 and 647 nm; (H) γ-proteobacteria probe; (I) α-proteobacteria probe; (N) Planctomycete probe; (E), (J), (O), and (T) merged image of (A-D), (F-I), (K-N), and (P-S); (R-S) unstained controls under three different channels for DAPI, 488 and 647 nm. Scale bars = 5 μm for A-E and K-T; 20 μm for F-J.
(PDF)

S3 Fig Summary of shared orthologs among vMAGS and reference genomes. (A) Upset plot showing shared number of ortholog clusters among vMAGs, reference genomes and RCC257 viral-subset-scaffolds. (B) Heatmap showing shared number of recruited scaffolds from RCC257 viral-subset-scaffolds for each genome. Red colour indicates more shared numbers of scaffolds to assemble vMAGs. OV_vMAG = combined orthologs predicted from OlVs-, OtV1-, OmV1-vMAGs; BV_vMAGs = combined orthologs predicted from BpVs-, BIIVs-vMAGs; BV-genomes = combines orthologs predicted from reference genomes of BpVs and BIIVs; OV_genomes = combined orthologs predicted from reference genomes of OlVs, OtV1 and OmV1; RCC257_subset_scaffolds = RCC257 viral-subset-scaffolds.
(PDF)

S4 Fig A multi-gene prasinovirus phylogeny reconstructed from 22 prasinovirus core genes. The phylogenetic tree consisting of 5,355 sites was reconstructed using IQ-TREE2 LG+F+G4 model, including genes searched from WGA data of two different *S*. *scintillans* strains, RCC24 and RCC257. The right panel shows presence-absence of select core genes. Single-copy genes are DNApol (DNA polymerase B), DNAhel-SNF2 (SNF2 helicase), mRNAcap (mRNA capping enzyme), ATPase, and RNR-sm (RNR small subunit). The tree is rooted with Chlorovirus (PBCVs and ATCV) for visualization. Only nodes <100% ultrafast bootstrap supports are labelled. OlV = *Ostreococcus lucimarinus* virus; OtV = *Ostreococcus tauri* virus; OmV = *Ostreococcus mediterraneus* virus; MpV = *Micromonas pusilla* virus; BpV = *Bathycoccus prasinos* virus; BIIV = *Bathycoccus* sp. virus clade BII. PBCV = *Paramecium bursaria chlorella* virus; ATCV = *Acanthocystis turfaceae chlorella* virus.
(PDF)

S5 Fig Genome overview and comparison of select vMAGs to corresponding reference genomes. (A) Circularized representation of (A) RCC24 BpV-vMAG compared to BpV2 genome and RCC257 BpV-vMAG; (B) OlV2 genome compared to RCC24 and RCC257 OlV2-vMAGs, (C) MpV-Pl1 genome compared to RCC24 and RCC257 MpVPl1-vMAGS, in an ordered set of coding sequences, represented by blocks shaded by similarity. (A) Mapping coverage is based on RCC24 BpV-vMAG mapped to RCC24 WGA viral-subset-scaffolds and regions with the coverage more than one standard deviation [59.9] from the mean coverage [44.5] are shown in blue spikes. The outermost ring represents predicted ORFs of the vMAG with manually annotated protein from Prodigal-gv and Viralrecall. (B) Mapping coverage is based on OlV2 genome mapped to RCC24 WGA viral-subset-scaffolds and regions with the coverage more than one standard deviation [5.1] from the mean coverage [1.6] shown in blue spikes. Only ORFs from the reference OlV2 genome is shown and the partial RCC24 and RCC257 OlV2-vMAG CDS are shown in the outer rings. (C) Mapping coverage is based on MpV-Pl1 mapped to RCC24 WGA viral-subset-scaffolds and regions with the coverage more than one standard deviation [2.1] from the mean coverage [0.6] are shown in blue spikes. Only ORFs from the reference MpV-Pl1 genome is shown and the partial RCC24 and RCC257 MpVPl1-vMAGs CDS are shown in the outer rings. See S2 Table for annotation in a tabular format.
(PDF)

S1 Dataset Summary annotations of BpV-vMAGs and BV-vMAG gene contents. (XLS)

S2 Dataset BlobToolKit Summary of RCC257 WGA sequencing results. (XLS)
