# Supplementary material for: Genomic analyses of Symbiomonas scintillans show no evidence for endosymbiotic bacteria but does reveal the presence of giant viruses
Source: PLoS Genet. 2024 Apr 1;20(4):e1011218. doi: 10.1371/journal.pgen.1011218 (PMC11008856; doi:10.1371/journal.pgen.1011218)
Supplement: S1 Table — RCC = Roscoff Culture Collection, France; NIES = Microbial Culture Collection at the National Institute of Environmental Studies, Japan; WGA = Whole genome amplification; AF-SMG = Amplification-free shotgun metagenome. (DOCX) [file pgen.1011218.s001.docx]

**S1 Table.** **Summary of different strains and sequencing data of *Symbiomonas scintillans* examined in this study**.

|  | **Culture collection** | **Isolation location** | **FISH location** | **Sequencing method** | **Sequencing location** | **Library Prep Date (M/Y)** | **Reported strain in**  **Guillou et al., 1999 [17]** |
| --- | --- | --- | --- | --- | --- | --- | --- |
| RCC24 | RCC | Pacific Ocean | Canada | WGA | Canada | 04-2022 | Yes |
| RCC257 | RCC | Atlantic Ocean | Canada | WGA | Canada | 04-2022 | No |
| RCC257-late | RCC | Atlantic Ocean | Canada | WGA | Canada | 06-2022 | No |
| RCC257-jp | RCC | Atlantic Ocean | Japan | —— | —— | —— | No |
| RCC24-jp (NIES-2589) | NIES | Pacific Ocean | Japan | AF-SMG | Japan | 03,05,06,  10-2022 | Yes |
| RCC25 | RCC | Mediterranean Sea | —— | One of the two strains reported by Guillou et al., in 1999[17].  Lost at RCC in 2008 | | | |

RCC=Roscoff Culture Collection, France; NIES=Microbial Culture Collection at the National Institute of Environmental Studies, Japan; WGA=Whole genome amplification; AF-SMG=Amplification-free shotgun metagenome.
