# Supplementary material for: Genomic analyses of Symbiomonas scintillans show no evidence for endosymbiotic bacteria but does reveal the presence of giant viruses
Source: PLoS Genet. 2024 Apr 1;20(4):e1011218. doi: 10.1371/journal.pgen.1011218 (PMC11008856; doi:10.1371/journal.pgen.1011218)
Supplement: S2 Table — CheckV% indicates completeness for each vMAG assemblies. (DOCX) [file pgen.1011218.s002.docx]

**S2 Table. Summary of genomic characteristics and references of prasinoviruses used to guide genome assemblies and subsequent vMAGs from RCC24 and RCC257.**

| **Virus**  **Genomes** | **Length (bp)** | **GC %** | **ORFs** | **tRNAs** | **Genes** | **CheckV %** | **Accession** | **Publication** |
| --- | --- | --- | --- | --- | --- | --- | --- | --- |
| BIIV1 | 174,426 | 35.2 | 220 | 3 |  | NA | MK522034-MK522037 | [1] |
| BIIV2 | 207,870 | 36.5 | 235 | 2 | 249 | NA | MK522038 |  |
| BIIV3 | 211,597 | 36.3 | 230 | 3 | 241 | NA | MK522039 |  |
| BpV1 | 198,519 | 37.2 | 203 | 3 | 203 | NA | NC014765 | [2] |
| BpV2 | 187,069 | 37 | 210 | 4 | 225 | NA | HM004430 |  |
| OlV1 | 194,022 | 40.9 | 254 | 5 | 255 | NA | NC014766 | [3] |
| OlV2 | 196,300 | 41.2 | 269 | 5 | 274 | NA | NC028091 | [4] |
| OlV4 | 216,925 | 40.3 | 256 | 5 | 319 | NA | JF974316 |  |
| OlV5 | 186,468 | 41.6 | 254 | 4 | 263 | NA | NC020852 | [4] |
| OlV6 | 184,949 | 41.7 | 251 | 5 | 257 | NA | HQ633059 |  |
| OlV7 | 182,309 | 41 | 243 | 5 | 248 | NA | NC028093 | [3] |
| OmV1 | 193,301 | 44.6 | 252 | 5 | 257 | NA | NC028092 | [4] |
| OtV1 | 189,567 | 44.5 | 240 | 4 | 233 | NA | JN225873 |  |
| MpV1 | 184,095 | 39 | 244 | 6 | 244 | NA | NC014767 | [2] |
| MpV_12T | 205,622 | 39.8 | 253 | 7 | 265 | NA | NC020864 |  |
| MpV_Pl1 | 197,060 | 43.3 | 259 | 5 | 270 | NA | HQ633072 | [5] |
| RCC257_vMAG_BIIV1 | 104,406 | 36.17 | 153 |  | 137 | 54.61 |  |  |
| RCC257_vMAG_BIIV2 | 98,732 | 35.35 | 138 |  | 130 | 51.51 |  |  |
| RCC257_vMAG_BIIV3 | 83,238 | 36.04 | 116 |  | 109 | 43.54 |  |  |
| RCC257_vMAG_BpV1 | 193,823 | 36.13 | 295 | 2 | 254 | 100 |  |  |
| RCC257_vMAG_BpV2 | 195,514 | 36.27 | 297 | 2 | 259 | 100 |  |  |
| RCC257_vMAG_MpV1 | 19,211 | 39.79 | 23 |  | 21 | 10.03 |  |  |
| RCC257_vMAG_MpV12T | 15,031 | 40.58 | 15 |  | 15 | 7.85 |  |  |
| RCC257_vMAG_MpVPl1 | 34,707 | 41.52 | 47 |  | 40 | 18.11 |  |  |
| RCC257_vMAG_OlV1 | 102,789 | 39.97 | 149 |  | 146 | 53.93 |  |  |
| RCC257_vMAG_OlV2 | 87,373 | 39.91 | 119 |  | 113 | 45.83 |  |  |
| RCC257_vMAG_OlV4 | 56,877 | 39.55 | 88 |  | 82 | 29.77 |  |  |
| RCC257_vMAG_OlV5 | 89,948 | 39.99 | 116 |  | 114 | 47.18 |  |  |
| RCC257_vMAG_OlV6 | 89,926 | 39.98 | 119 |  | 117 | 47.17 |  |  |
| RCC257_vMAG_OlV7 | 102,138 | 40.01 | 152 |  | 143 | 53.59 |  |  |
| RCC257_vMAG_OmV1 | 67,431 | 39.84 | 811 |  | 71 | 35.37 |  |  |
| RCC257_vMAG_OtV1 | 68 | 39.7 | 85 | 1 | 73 | 35.89 |  |  |
| RCC24_vMAG_BIIV1 | 125,432 | 37.12 | 183 | 4 | 165 | 65.61 |  |  |
| RCC24_vMAG_BIIV2 | 97,551 | 37.27 | 113 | 3 | 108 | 51.03 |  |  |
| RCC24_vMAG_BIIV3 | 90,981 | 37.68 | 112 | 3 | 105 | 47.6 |  |  |
| RCC24_vMAG_BpV1 | 223,996 | 36.51 | 341 | 4 | 291 | 100 |  |  |
| RCC24_vMAG_BpV2 | 221,235 | 36.53 | 338 | 4 | 286 | 100 |  |  |
| RCC24_vMAG_MpV1 | 40,992 | 40.41 | 64 | 1 | 60 | 21.4 |  |  |
| RCC24_vMAG_MpVPl1 | 80,383 | 40.7 | 137 | 1 | 132 | 41.98 |  |  |
| RCC24_vMAG_OlV1 | 123,654 | 40.18 | 221 | 2 | 190 | 64.54 |  |  |
| RCC24_vMAG_OlV2 | 149,739 | 39.93 | 244 | 2 | 220 | 78.52 |  |  |
| RCC24_vMAG_OlV4 | 72,632 | 40.29 | 125 |  | 119 | 38.1 |  |  |
| RCC24_vMAG_OlV5 | 121,783 | 40.15 | 196 | 2 | 188 | 63.87 |  |  |
| RCC24_vMAG_OlV6 | 119,913 | 40.14 | 196 | 2 | 182 | 62.59 |  |  |
| RCC24_vMAG_OlV7 | 120,063 | 40.46 | 203 | 2 | 176 | 62.69 |  |  |
| RCC24_vMAG_OmV1 | 125,261 | 39.89 | 196 | 2 | 186 | 65.39 |  |  |
| RCC24_vMAG_OtV1 | 92,373 | 39.38 | 137 |  | 121 | 48.43 |  |  |

CheckV% indicates completeness for each vMAG assemblies.

**References**

1. Bachy C, Yung CCM, Needham DM, Gazitúa MC, Roux S, Limardo AJ, et al. Viruses infecting a warm water picoeukaryote shed light on spatial co-occurrence dynamics of marine viruses and their hosts. ISME J. 2021;15: 3129–3147. doi:10.1038/s41396-021-00989-9

2. Moreau H, Piganeau G, Desdevises Y, Cooke R, Derelle E, Grimsley N. Marine Prasinovirus Genomes Show Low Evolutionary Divergence and Acquisition of Protein Metabolism Genes by Horizontal Gene Transfer. J Virol. 2010;84: 12555–12563. doi:10.1128/JVI.01123-10

3. Zimmerman AE, Bachy C, Ma X, Roux S, Jang HB, Sullivan MB, et al. Closely related viruses of the marine picoeukaryotic alga *Ostreococcus lucimarinus* exhibit different ecological strategies. Environ Microbiol. 2019;21: 2148–2170. doi:10.1111/1462-2920.14608

4. Derelle E, Monier A, Cooke R, Worden AZ, Grimsley NH, Moreau H. Diversity of Viruses Infecting the Green Microalga Ostreococcus lucimarinus. Sandri-Goldin RM, editor. J Virol. 2015;89: 5812–5821. doi:10.1128/JVI.00246-15

5. Finke J, Winget D, Chan A, Suttle C. Variation in the Genetic Repertoire of Viruses Infecting Micromonas pusilla Reflects Horizontal Gene Transfer and Links to Their Environmental Distribution. Viruses. 2017;9: 116. doi:10.3390/v9050116
