# Supplementary figures and images for: Genomic analyses of Symbiomonas scintillans show no evidence for endosymbiotic bacteria but does reveal the presence of giant viruses
Source: PLoS Genet. 2024 Apr 1;20(4):e1011218. doi: 10.1371/journal.pgen.1011218 (PMC11008856; doi:10.1371/journal.pgen.1011218)

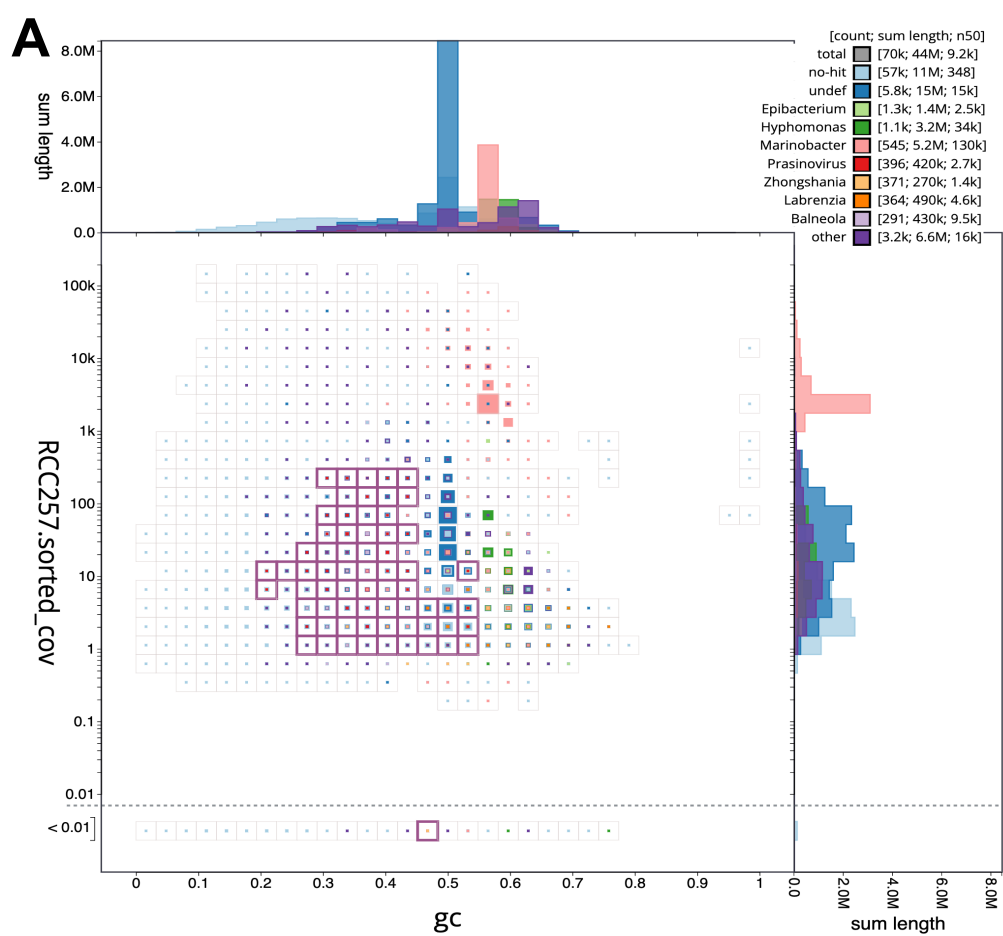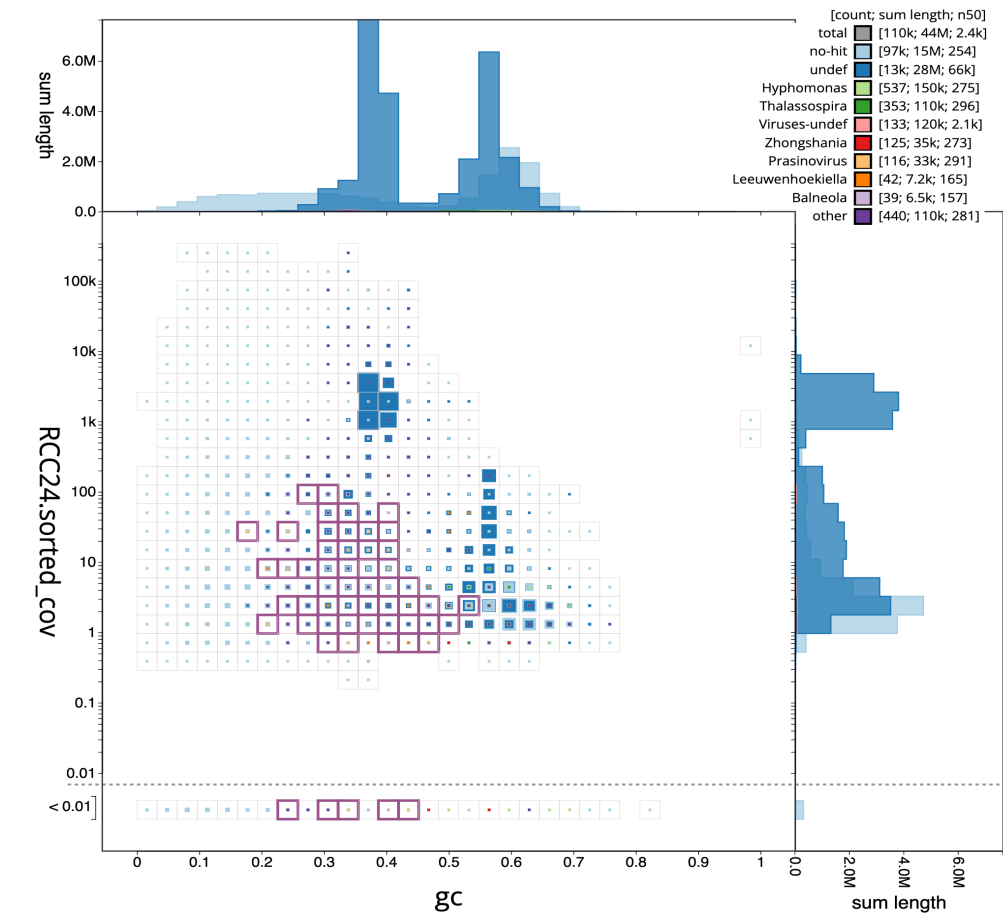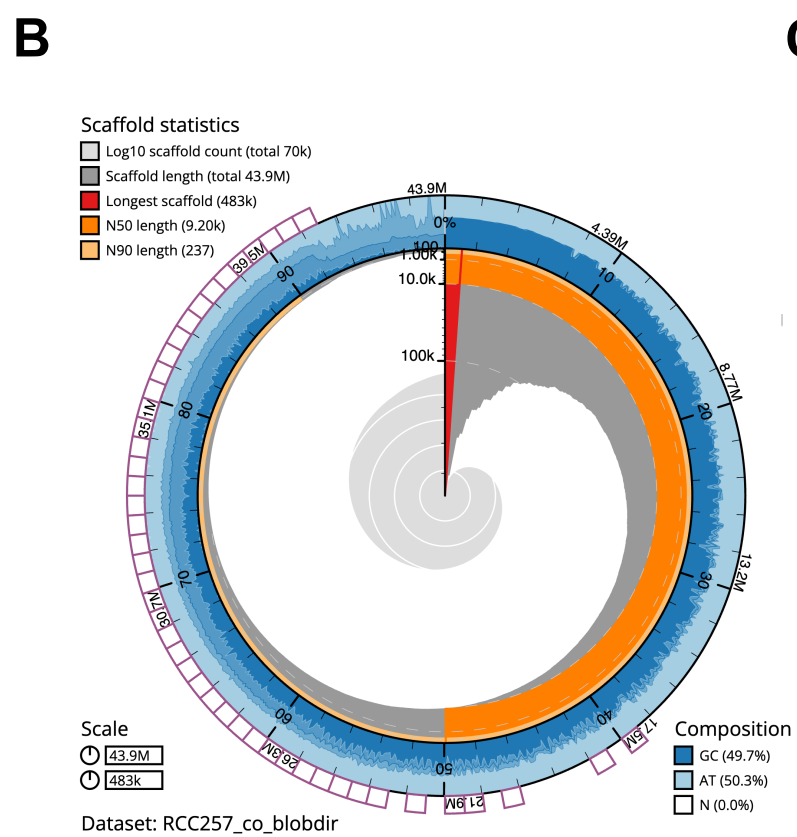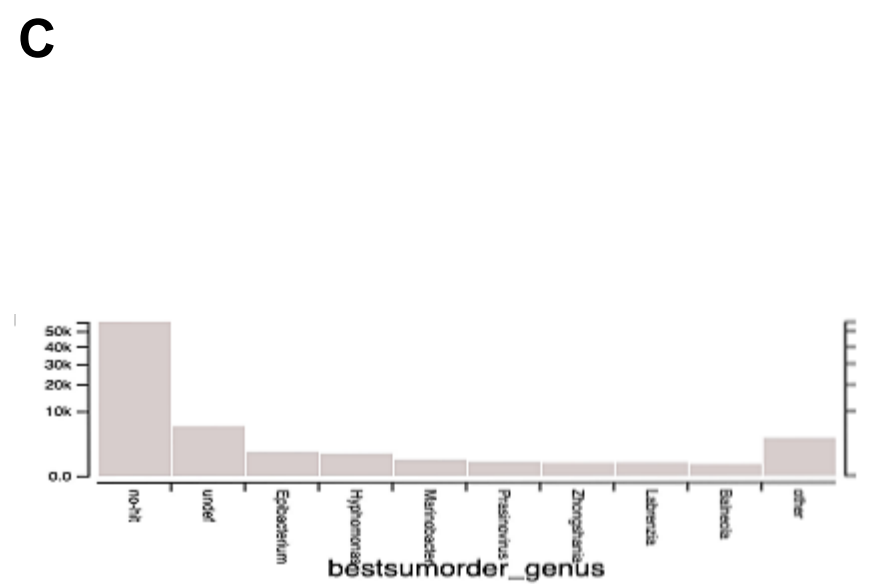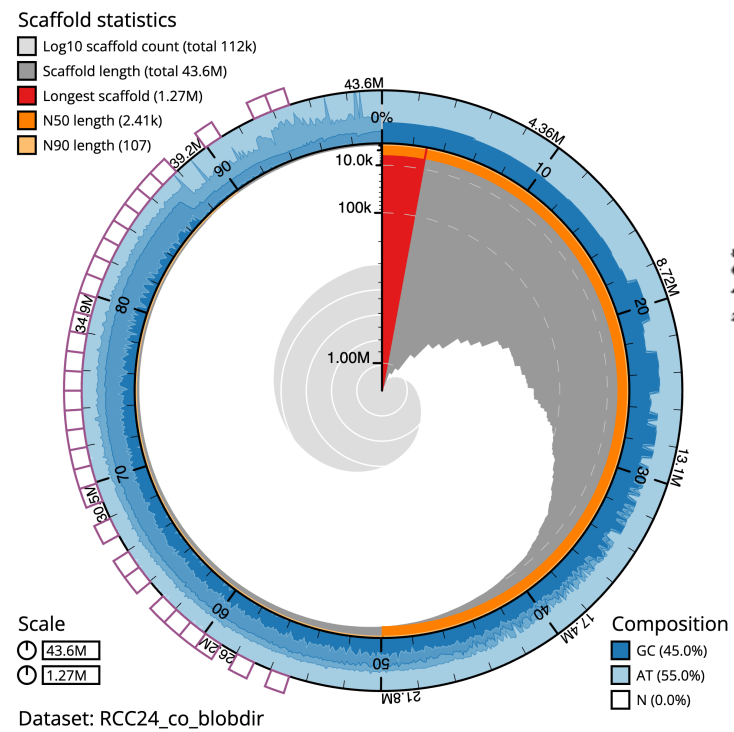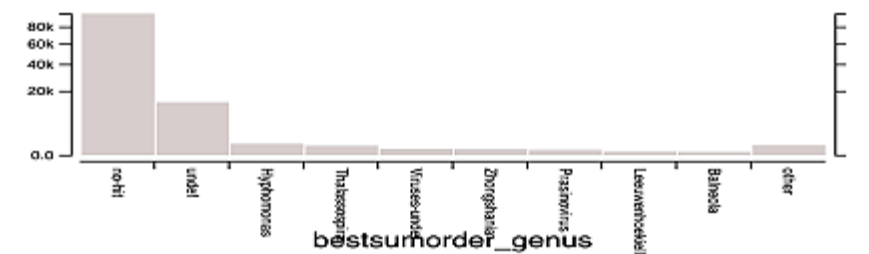

Supplement: S1 Fig — (A) Blob plots based on mean coverage (per-base) in y-axis and mean GC contents in x-axis. Each “blob” represents a square-root scaled size (showing max size) of a scaffold with its size representing the length or span. The blobs are coloured according to the top ten taxonomic assignment at the genus level (‘bestsum’ taxrule), based on coverage. Sum lengths along each axis are plotted on histograms. All reads assigned to prasinoviruses are highlighted with purple squares around each blob. (B) Snail plots visualizing quality of the initial assembly represented by N50 and N90. The purple squares in the blob plots and ones positioned at the outermost part of the plots are scaffolds assigned to prasinoviruses. (C) Histograms showing coverage (y-axis) for top ten genus (including “no-hit”, “undefined” and “others”). (PDF) [file pgen.1011218.s003.pdf]

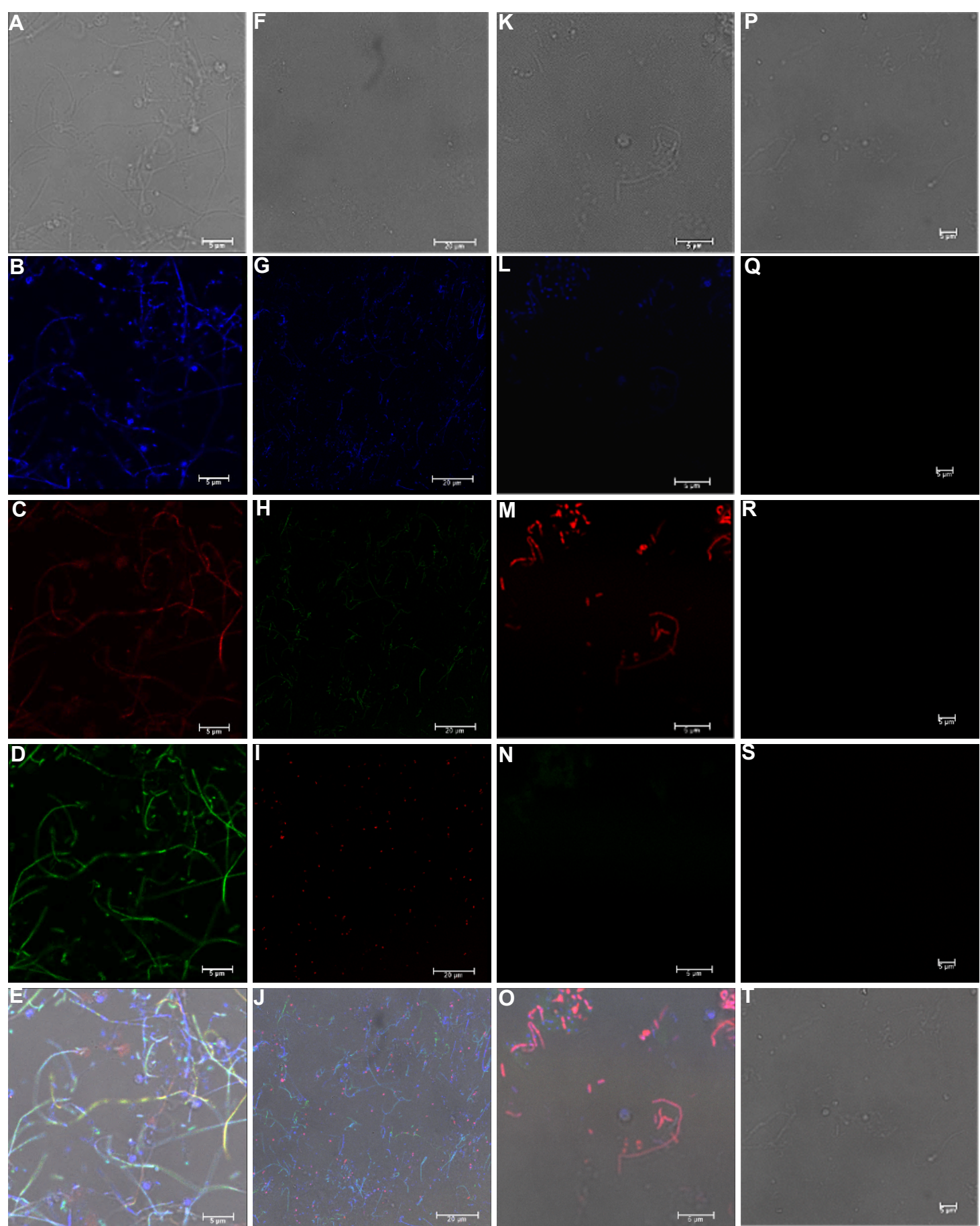

Supplement: S2 Fig — (A), (F), (K), and (P) Brightfield; (B), (G), and (L) DAPI; (C) CF319 probe under 647 nm; (D) and (M) EUB388 probe under 488 and 647 nm; (H) γ-proteobacteria probe; (I) α-proteobacteria probe; (N) Planctomycete probe; (E), (J), (O), and (T) merged image of (A-D), (F-I), (K-N), and (P-S); (R-S) unstained controls under three different channels for DAPI, 488 and 647 nm. Scale bars = 5 μm for A-E and K-T; 20 μm for F-J. (PDF) [file pgen.1011218.s004.pdf]

**A**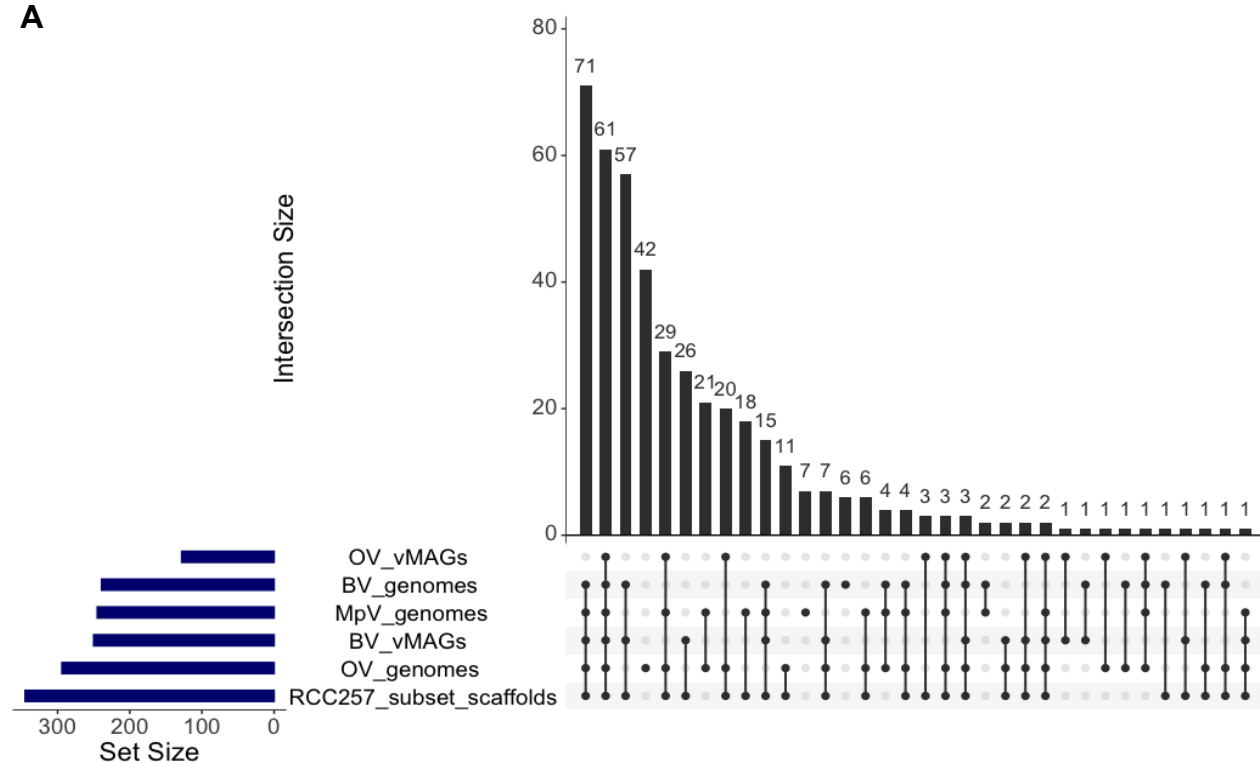**B**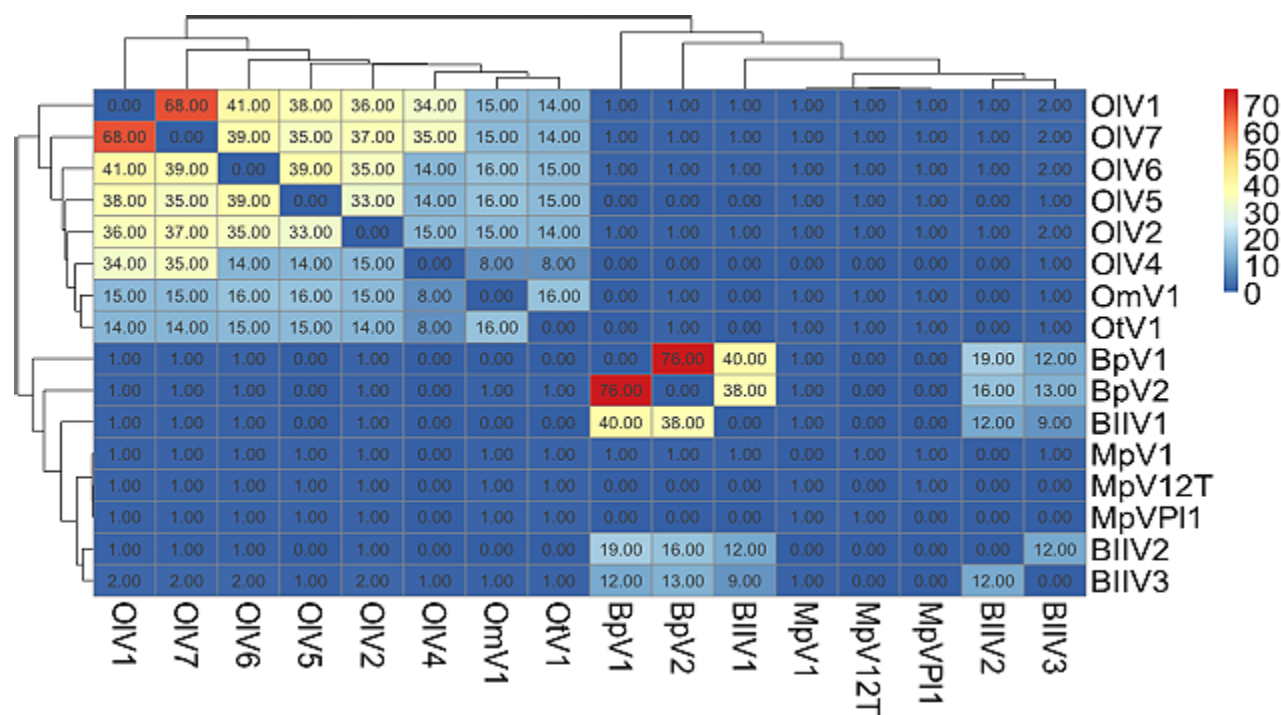

Supplement: S3 Fig — (A) Upset plot showing shared number of ortholog clusters among vMAGs, reference genomes and RCC257 viral-subset-scaffolds. (B) Heatmap showing shared number of recruited scaffolds from RCC257 viral-subset-scaffolds for each genome. Red colour indicates more shared numbers of scaffolds to assemble vMAGs. OV_vMAG = combined orthologs predicted from OlVs-, OtV1-, OmV1-vMAGs; BV_vMAGs = combined orthologs predicted from BpVs-, BIIVs-vMAGs; BV-genomes = combines orthologs predicted from reference genomes of BpVs and BIIVs; OV_genomes = combined orthologs predicted from reference genomes of OlVs, OtV1 and OmV1; RCC257_subset_scaffolds = RCC257 viral-subset-scaffolds. (PDF) [file pgen.1011218.s005.pdf]

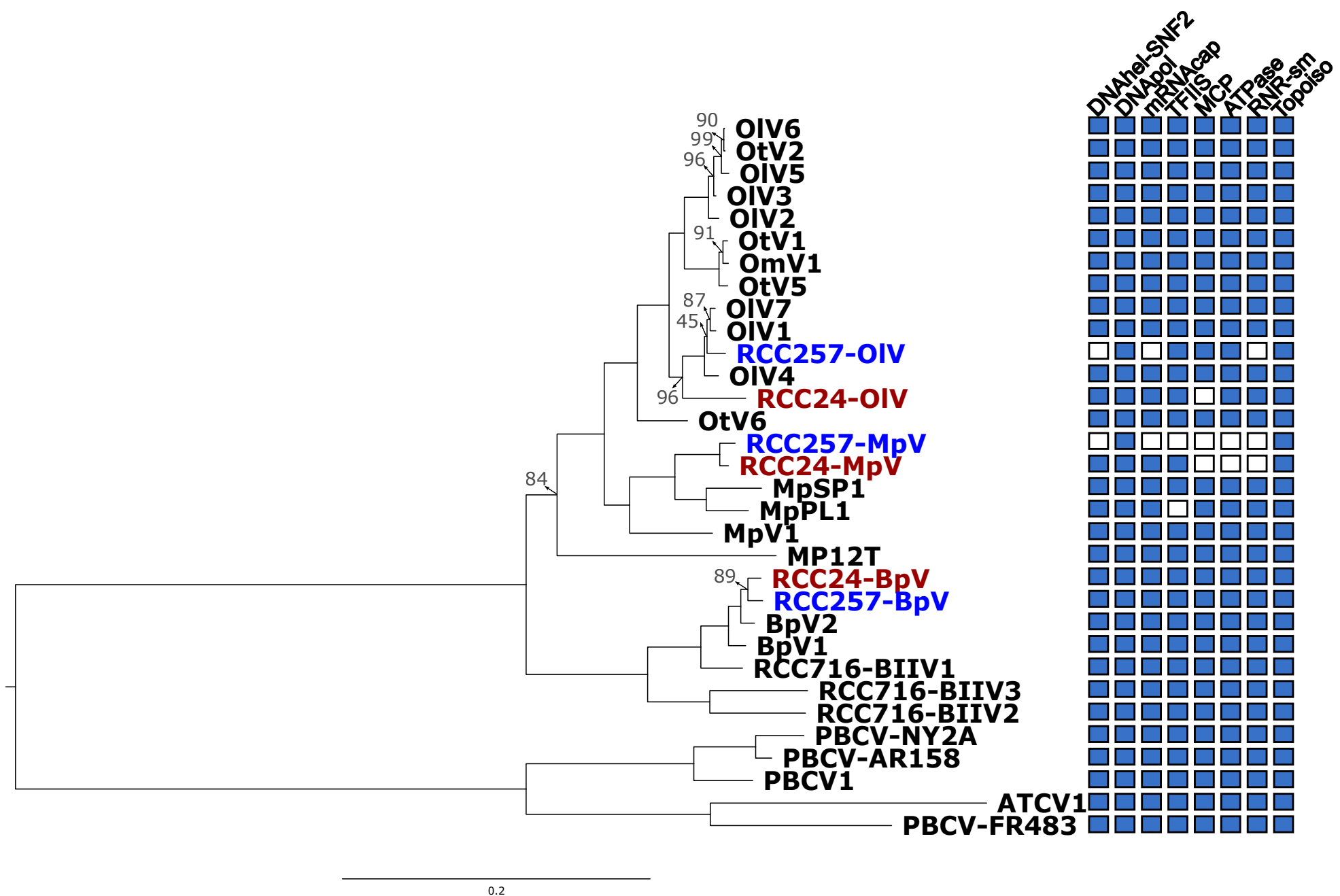

Supplement: S4 Fig — The phylogenetic tree consisting of 5,355 sites was reconstructed using IQ-TREE2 LG+F+G4 model, including genes searched from WGA data of two different S. scintillans strains, RCC24 and RCC257. The right panel shows presence-absence of select core genes. Single-copy genes are DNApol (DNA polymerase B), DNAhel-SNF2 (SNF2 helicase), mRNAcap (mRNA capping enzyme), ATPase, and RNR-sm (RNR small subunit). The tree is rooted with Chlorovirus (PBCVs and ATCV) for visualization. Only nodes <100% ultrafast bootstrap supports are labelled. OlV = Ostreococcus lucimarinus virus; OtV = Ostreococcus tauri virus; OmV = Ostreococcus mediterraneus virus; MpV = Micromonas pusilla virus; BpV = Bathycoccus prasinos virus; BIIV = Bathycoccus sp. virus clade BII. PBCV = Paramecium bursaria chlorella virus; ATCV = Acanthocystis turfaceae chlorella virus. (PDF) [file pgen.1011218.s006.pdf]

**A**

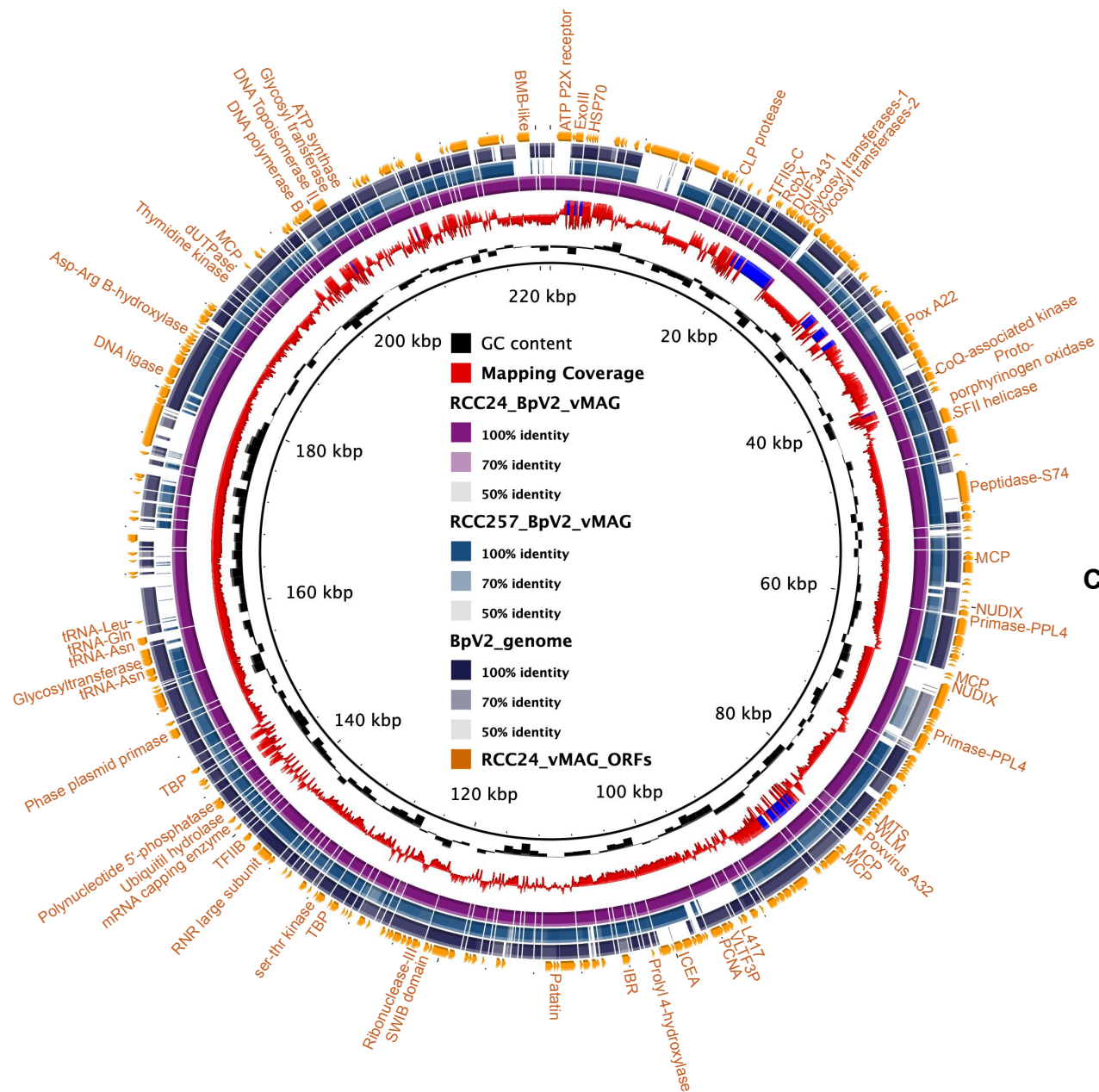

**B**

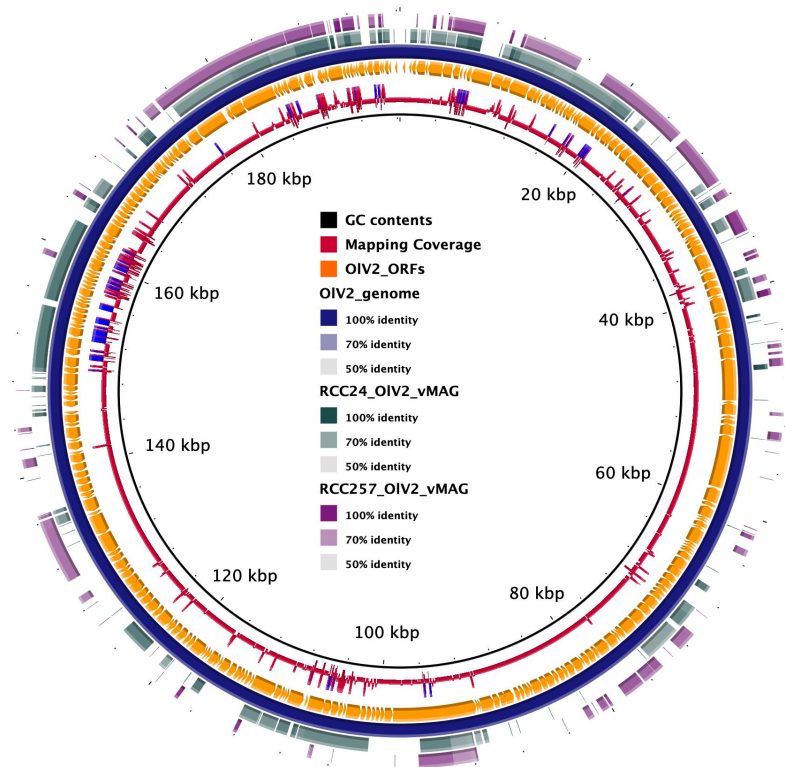

C

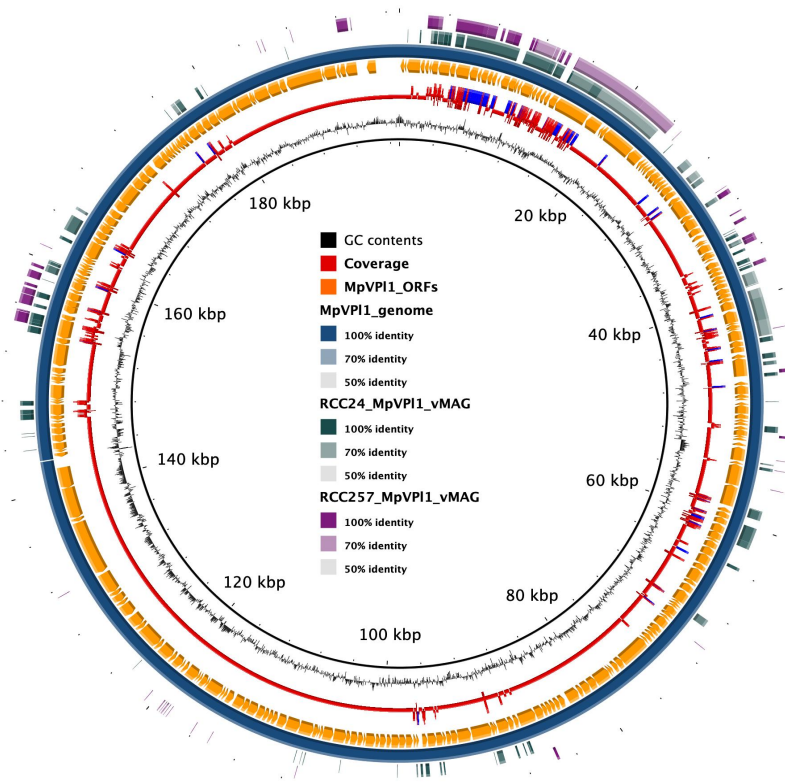

Supplement: S5 Fig — (A) Circularized representation of (A) RCC24 BpV-vMAG compared to BpV2 genome and RCC257 BpV-vMAG; (B) OlV2 genome compared to RCC24 and RCC257 OlV2-vMAGs, (C) MpV-Pl1 genome compared to RCC24 and RCC257 MpVPl1-vMAGS, in an ordered set of coding sequences, represented by blocks shaded by similarity. (A) Mapping coverage is based on RCC24 BpV-vMAG mapped to RCC24 WGA viral-subset-scaffolds and regions with the coverage more than one standard deviation [59.9] from the mean coverage [44.5] are shown in blue spikes. The outermost ring represents predicted ORFs of the vMAG with manually annotated protein from Prodigal-gv and Viralrecall. (B) Mapping coverage is based on OlV2 genome mapped to RCC24 WGA viral-subset-scaffolds and regions with the coverage more than one standard deviation [5.1] from the mean coverage [1.6] shown in blue spikes. Only ORFs from the reference OlV2 genome is shown and the partial RCC24 and RCC257 OlV2-vMAG CDS are shown in the outer rings. (C) Mapping coverage is based on MpV-Pl1 mapped to RCC24 WGA viral-subset-scaffolds and regions with the coverage more than one standard deviation [2.1] from the mean coverage [0.6] are shown in blue spikes. Only ORFs from the reference MpV-Pl1 genome is shown and the partial RCC24 and RCC257 MpVPl1-vMAGs CDS are shown in the outer rings. See S2 Table for annotation in a tabular format. (PDF) [file pgen.1011218.s007.pdf]
